# Variation-preserving normalization unveils blind spots in gene expression profiling

**DOI:** 10.1038/srep42460

**Published:** 2017-03-09

**Authors:** Carlos P. Roca, Susana I. L. Gomes, Mónica J. B. Amorim, Janeck J. Scott-Fordsmand

**Affiliations:** 1 Department of Chemical Engineering, Universitat Rovira i Virgili, 43007 Tarragona, Spain; 2 Department of Bioscience, Aarhus University, 8600 Silkeborg, Denmark; 3 Department of Biology & CESAM, University of Aveiro, 3810-193 Aveiro, Portugal

## Abstract

RNA-Seq and gene expression microarrays provide comprehensive profiles of gene activity, but lack of reproducibility has hindered their application. A key challenge in the data analysis is the normalization of gene expression levels, which is currently performed following the implicit assumption that most genes are not differentially expressed. Here, we present a mathematical approach to normalization that makes no assumption of this sort. We have found that variation in gene expression is much larger than currently believed, and that it can be measured with available assays. Our results also explain, at least partially, the reproducibility problems encountered in transcriptomics studies. We expect that this improvement in detection will help efforts to realize the full potential of gene expression profiling, especially in analyses of cellular processes involving complex modulations of gene expression.

Since the discovery of DNA structure by Watson and Crick, molecular biology has progressed increasingly fast, with rapid advances in sequencing and related genomic technologies. Among these, DNA microarrays and RNA-Seq have been widely adopted to obtain gene expression profiles, by measuring the concentration of tens of thousands of mRNA molecules in single assays^1^,^2^,^3^,^4^,^5^. Despite their enormous potential^6^,^7^,^8^,^9^, problems of reproducibility and reliability^10^,^11^,^12^ have discouraged their use in some areas, e.g. biomedicine^13^,^14^,^15^,^16^.

The normalization of gene expression, which is required to set a common reference level among samples^17^,^18^,^19^,^20^, has been reported to be problematic, affecting the reproducibility of results with both microarray^21^,^22^,^23^ and RNA-Seq^24^,^25^,^26^,^27^. Batch effects and their influence on normalization have recently received a great deal of attention^28^,^29^,^30^, resulting in approaches aiming to remove unwanted technical variation caused by differences between batches of samples or by other sources of expression heterogeneity^31^,^32^,^33^. A different issue, however, is the underlying assumption made by the most widely used normalization methods to date, such as Median and Quantile normalization^34^ for microarrays, or RPKM (Reads Per Kilobase per Million mapped reads)^4^, TMM (Trimmed Mean of M-values)^35^, and DESeq^36^ normalization for RNA-Seq, which posit that all or most genes are not differentially expressed^25^,^37^,^38^,^39^,^40^. Although it may seem reasonable for many applications, this *lack-of-variation assumption* has not been confirmed. Moreover, results obtained with external controls^37^,^38^,^39^,^41^ or with RT-qPCR^21^,^24^ suggest that it may not be valid.

Some methods have been proposed to address this issue, based on the use of spike-ins^37^,^38^,^39^, negative control probes (SQN, Subset Quantile normalization)^42^, or negative control genes (RUV-2, Remove Unwanted Variation, 2-step)^32^. These methods use external or internal controls that are *known a priori* not to be differentially expressed^43^. Their applicability, however, has been limited by this requirement of a priori knowledge, which is rarely available for a sufficiently large number of controls. In addition, other methods have been proposed to address the lack-of-variation assumption by identifying a subset of non-differentially expressed genes from the assay data, such as Cross-Correlation normalization^44^, LVS (Least-Variant Set) normalization^45^, and NVAS (Nonparametric Variable Selection and Approximation) normalization^46^. While LVS normalization requires setting in advance a number for the fraction of genes to be considered as non-differentially expressed, with values in the range 40–60%^45^, Cross-Correlation and NVAS normalization are expected to degrade in performance when more than 50% of genes are differentially expressed^44^,^46^. More recently, CrossNorm has been introduced^47^, based on the mixture of gene expression distributions from the experimental conditions. This method, however, has been proposed for two experimental conditions, and specially for paired samples. The extension of this approach to experimental designs with unpaired samples and more than a few experimental conditions would lead, as far as we can hypothesize, to an unmanageable size of the data matrix to process.

Thus, to clarify and overcome the limitations imposed by the lack-of-variation assumption, we have developed an approach to normalization that does not assume lack-of-variation and that is suitable to most real-world applications. Hence, we aimed to avoid the need of spike-ins, a priori knowledge of control genes, or assumptions on the number of differentially expressed genes. The analysis of several gene expression datasets using this approach confirmed that our methods reached these goals. Furthermore, our results show that assuming lack-of-variation can severely undermine the detection of gene expression variation in real assays. We have found that large numbers of differentially expressed genes, with substantial expression changes, are missed or misidentified when data are normalized with methods that assume lack-of-variation.

## Results

### *E. crypticus* and Synthetic Datasets

A large gene expression dataset was obtained from biological triplicates of *Enchytraeus crypticus* (a globally distributed soil organism used in standard ecotoxicity tests), sampled under 51 experimental conditions (42 treatments and 9 controls), involving exposure to several substances, at several concentrations and durations according to a factorial design (Supplementary Table S1). Gene expression was measured using a customized high-density oligonucleotide microarray^48^, resulting in a dataset with 18,339 gene probes featuring good hybridization signal in all 153 samples. Taking into account the design of the microarray^48^, we refer to these gene probes as *genes* in what follows.

To further explore and compare outcomes between normalization methods, two synthetic random datasets were built and analyzed. One of them was generated with identical means and variances gene-by-gene to the real *E. crypticus* dataset, and under the assumption that no gene was differentially expressed. In addition, normalization factors were applied, equal to those obtained from the real dataset. Thus, this synthetic dataset was similar to the real one, while complying by construction with the lack-of-variation assumption. The other synthetic dataset was also generated with comparable means and variances to the real dataset and with normalization factors, but in this case differential expression was added. Depending on the experimental condition, several numbers of differentially expressed genes and ratios between over- and under-expressed genes were introduced (see Methods). Together, these synthetic datasets with and without differential gene expression represent, respectively, the alternative and null hypotheses for a statistical test of differential gene expression.

### Normalization Methods

The gene expression datasets were normalized with four methods. Two of these methods are the most widely used procedures for microarrays, namely Median (or Scale) normalization and Quantile normalization^34^. (Note that current methods of normalization for RNA-Seq, such as RPKM^4^, TMM^35^, and DESeq^36^, perform between-sample normalization by introducing a scaling per sample obtained with some form of mean or median, using all or a large set of genes. Thus their performance, in what concerns the issues addressed here, is expected to be similar to that of Median normalization for microarrays.)

The other two normalization methods were developed for this study, they being called *Median Condition-Decomposition normalization* and *Standard-Vector Condition-Decomposition normalization*, respectively MedianCD and SVCD normalization in what follows.

With the exception of Quantile normalization, all used methods apply a multiplicative factor to the expression levels of each sample, equivalent to the addition of a number in the usual log_2_-scale for gene expression levels. Solving the *normalization problem* consists of finding these correction factors. This problem can be exactly and linearly decomposed into several sub-problems: one within-condition normalization for each experimental condition and one final between-condition normalization for the condition averages (see Methods). In the within-condition normalizations, the samples (replicates) subjected to each experimental condition are normalized separately, whereas in the final between-condition normalization average levels for all conditions are normalized together. Because there are no genes with differential expression in any of the within-condition normalizations, the lack-of-variation assumption only affects the final between-condition normalization. The assumption is avoided by using, in this normalization, expression levels only from *no-variation genes*, defined as genes that show no evidence of differential expression under a statistical test. An important detail is that the within-condition normalizations ensure good estimates of the within-condition variances, which are required by the statistical test for identifying no-variation genes. This requisite also implies that a minimum of two samples is required per experimental condition. Both methods of normalization proposed here, MedianCD and SVCD normalization, follow this condition-decomposition approach.

With MedianCD normalization, all normalizations are performed with median values, as in conventional Median normalization, but only no-variation genes are employed in the between-condition step. Otherwise, if all genes were used in this final step, the resulting total normalization factors would be exactly the same as those obtained with conventional Median normalization.

For SVCD normalization, a vectorial procedure was developed to carry out each normalization step, called Standard-Vector normalization. The samples of any experimental condition, in a properly normalized dataset, must be *exchangeable*. In mathematical terms, the expression levels of each gene can be considered as an *s*-dimensional vector, where *s* is the number of samples for the experimental condition. After standardization (mean subtraction and variance scaling), these standard vectors are located in a (*s* − 2)-dimensional hypersphere. The exchangeability mentioned above implies that, when properly normalized, the distribution of standard vectors must be invariant with respect to permutations of the samples and must have zero expected value. These properties allow to obtain a robust estimator of the normalization factors, under fairly general assumptions that do not imply any particular distribution of gene expression (see Methods).

It is worth mentioning that the limit case when the number of samples is two (*s* = 2) represents a degenerate case for Standard-Vector normalization, in which the space of standard vectors reduces to a 0-dimensional space with only two points. In this degenerate case, Standard-Vector normalization is equivalent to global Loess normalization^49^,^50^, i.e. Loess normalization without correction for non-linearities with respect to the level of gene expression or microarray print-tips. In this sense, Standard-Vector normalization is a generalization to any number of samples of the approach underlying the different types of Loess normalization.

### Normalization Results

**Figure 1:**
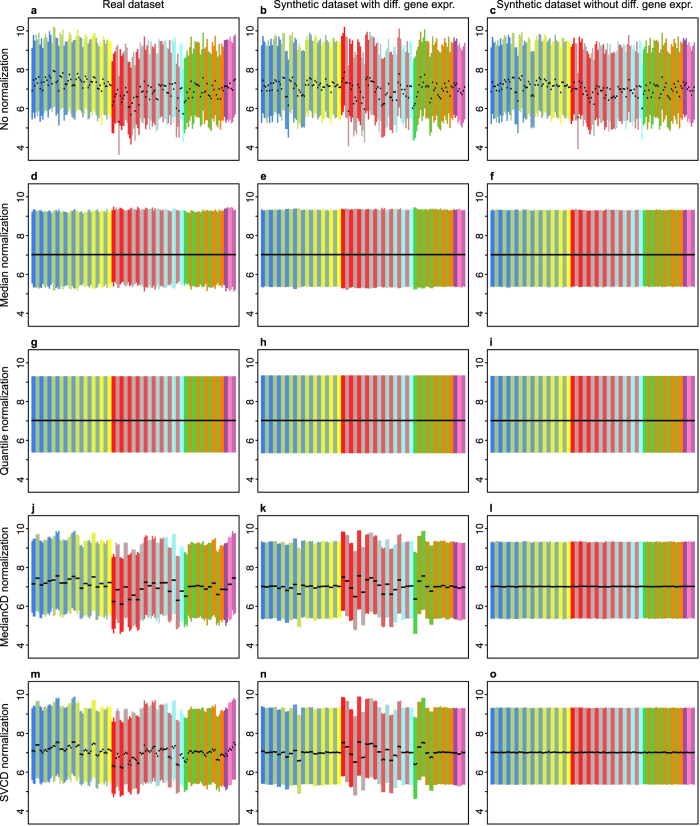
MedianCD and SVCD normalization resulted in the detection of much larger between-condition variation in the datasets with differential gene expression, compared to Median and Quantile normalization. Panels show interquartile ranges of expression levels for the 153 samples, grouped by the 51 experimental conditions (Ag, blue-yellow; Cu, red-cyan; Ni, green-orange; UV, purple; see Supplementary Table S1). Black lines indicate medians. Rows and columns correspond to normalization methods and datasets, respectively, as labeled.

Figure 1 displays the results of applying the four normalization methods to the real and two synthetic datasets. Each panel shows the interquartile range of expression levels for the 153 samples, grouped in triplicates exposed to each experimental condition. Both Median and Quantile normalization (second and third rows) yielded similar outputs for the three datasets. In contrast, MedianCD and SVCD normalization (fourth and fifth rows) detected much greater variation between conditions in the real dataset and the synthetic dataset with differential gene expression. Conventional Median normalization makes, by design, the median of all samples to be the same, while Quantile normalization makes the full distribution of gene expression of all samples to be the same. Hence, if there were differences in medians or distributions between experimental conditions, both methods would have removed them. Such variation was indeed present in the synthetic dataset with differential gene expression (Fig. 1k,n), and hence we can hypothesize the same for the real dataset (Fig. 1j,m).

### Influence of No-Variation Genes on Normalization

To clarify how MedianCD and SVCD normalization preserved the variation between conditions, we studied the influence of the choice of no-variation genes in the final between-condition normalization. To this end, we obtained the between-condition variation as a function of the number of no-variation genes, in two families of cases. In one family, no-variation genes were chosen in decreasing order of *p*-values from the statistical test used to analyze variation between conditions. In the other family, genes were chosen at random. The first option was similar to the approach implemented to obtain the results presented in Fig. 1j–o, with the difference that, there, the no-variation genes were chosen automatically, by a subsequent statistical test performed on the distribution of *p*-values (see Methods).

**Figure 2:**
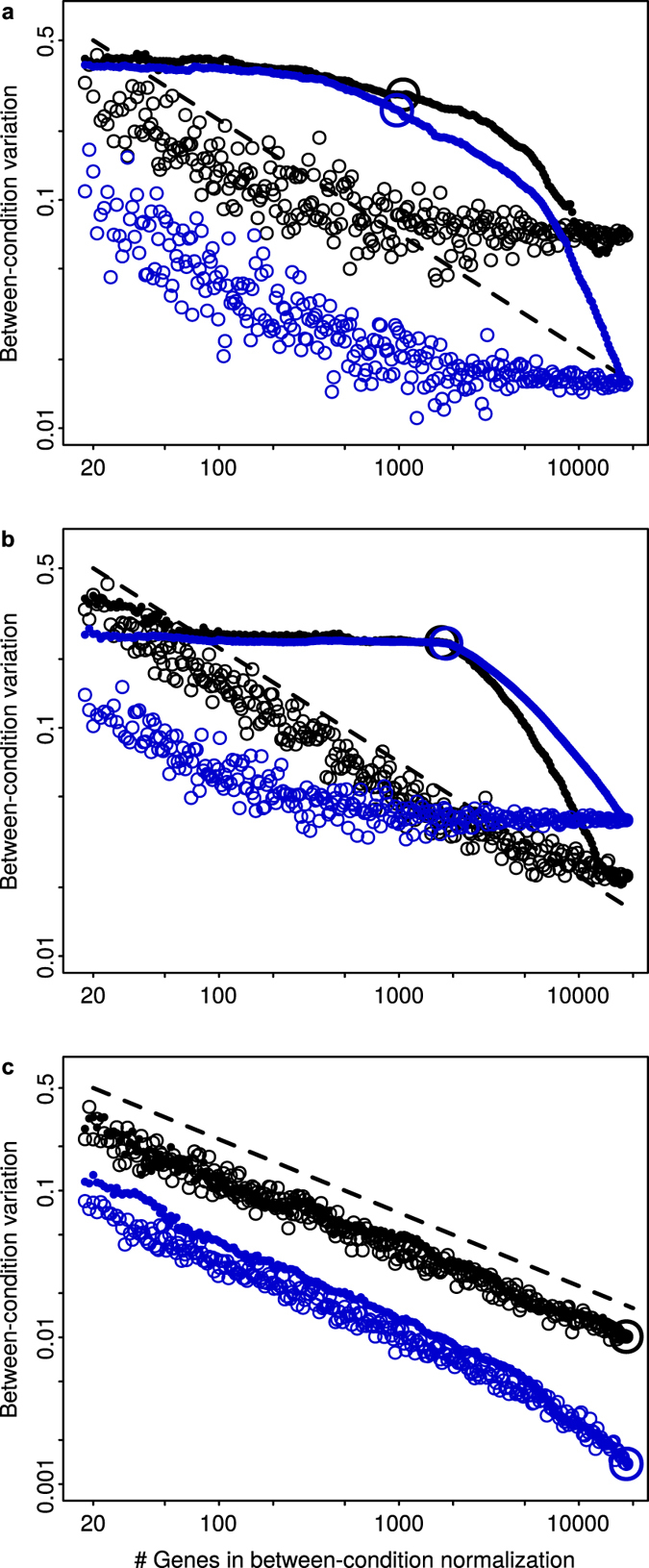
The selection of genes for the final between-condition normalization in MedianCD and SVCD normalization was crucial to preserve the variation between conditions. Panels show the detected variation as a function of the number of genes used in the between-condition normalization, for the real dataset (**a**), synthetic dataset with differential gene expression (**b**), and synthetic dataset without differential gene expression (**c**). Between-condition variation is represented as the standard deviation of the within-condition mean averages (averages of sample means, for all samples of the condition). See Supplementary Fig. S1 for results using within-condition median averages, with similar behavior. Each point in each panel indicates the variation obtained with one complete normalization (black circles, MedianCD normalization; blue circles, SVCD normalization). Genes were selected in two ways: randomly (empty circles) or in decreasing order of *p*-values from a test for detecting no-variation genes (filled circles). Big circles show the working points corresponding to the results depicted in Fig. 1j–o, which were chosen automatically. Black dashed lines show references for *n*^−1/2^ decays, with the same values in all panels.

For the real dataset (Fig. 2a), the random choice of genes resulted in *n*^−1/2^ decays (*n* being the number of chosen genes), followed by a plateau. The *n*^−1/2^ decays reflect the error in the estimation of normalization factors. Selecting the genes by decreasing *p*-values, however, yielded a completely different result. Up to a certain number of genes, the variance remained similar, but for larger numbers of genes the variance dropped rapidly. Figure 2a shows, therefore, that between-condition variation was removed as soon as the between-condition normalizations used genes that changed in expression level across experimental conditions. The big circles in Fig. 2a indicate the working points of the normalizations used for the results displayed in Fig. 1j,m. In fact, these points slightly underestimated the variation between conditions. Although the statistical test for identifying no-variation genes ensured that no evidence of variation was found, the expression of some selected genes varied across conditions.

The results obtained for the synthetic dataset with differential gene expression (Fig. 2b) were qualitatively similar to those of the real dataset, but with two important differences. The amount of between-condition variation detected (by selecting no-variation genes by decreasing *p*-values) was smaller than with the real dataset, implying that the real dataset had larger differential gene expression. Additionally, the variation detected in the synthetic dataset had a simpler dependency on the number of genes, an indication that the differential gene expression introduced in the synthetic dataset had a simpler structure than that of the real dataset.

Figure 2c shows the results for the synthetic dataset without differential gene expression. There were no plateaus when no-variation genes were chosen randomly, only *n*^−1/2^ decays, and differences were small when no-variation genes were selected by decreasing *p*-values. Big circles show that the working points of Fig. 1l,o were selected with all available genes as no-variation genes, which is the optimum choice when there is no differential gene expression.

Overall, Fig. 2 shows that the between-condition variation displayed in Fig. 1j,k,m,n is not an artifact caused by using an exceedingly small or extremely particular set of genes in the final between-condition normalization, but that this variation originated from the datasets. The positions of the big circles in Fig. 2 highlight the good performance of the statistical approach for choosing no-variation genes in the normalizations carried out for Fig. 1j–o. Besides, the residual variation displayed by the *n*^−1/2^ decays implies that, as estimators of the normalization factors, SVCD normalization features smaller error than MedianCD normalization.

### Differential Gene Expression

In what follows, we call *detected positives* the differentially expressed genes (DEGs) resulting from the statistical analyses, *treatment positives* the DEGs introduced in the synthetic dataset with differential gene expression, *true positives* the detected positives which were also treatment positives, and *false positives* the detected positives which were not treatment positives. Corresponding terms for *negatives* refer to genes which were not DEGs.

**Figure 3:**
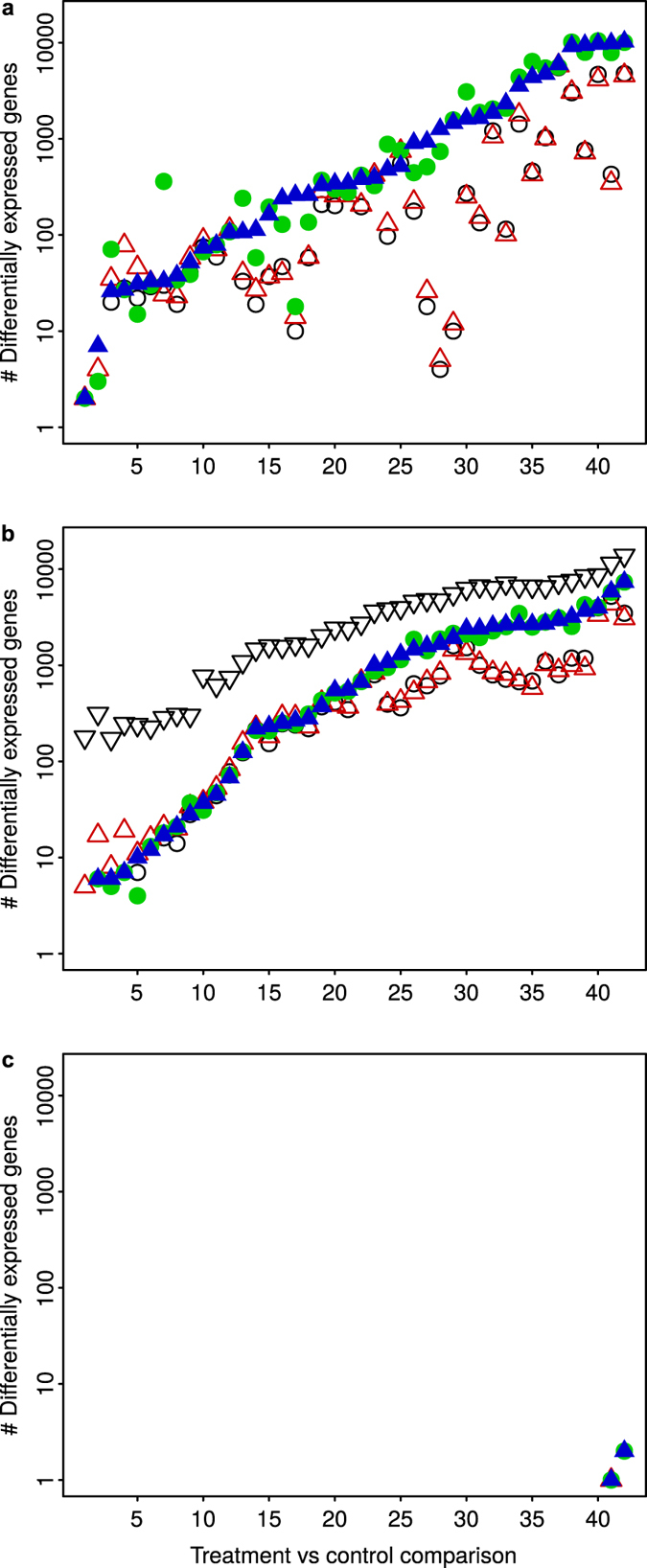
MedianCD and SVCD normalization allowed to detect much larger numbers of differentially expressed genes (DEGs) in the datasets with differential gene expression. Panels show results for the real dataset (**a**), synthetic dataset with differential gene expression (**b**), and synthetic dataset without differential gene expression (**c**). They display the number of DEGs for each treatment compared to the corresponding control, obtained after applying the four normalization methods (empty black circles, Median normalization; empty red up triangles, Quantile normalization; filled green circles, MedianCD normalization; filled blue up triangles, SVCD normalization). For the synthetic dataset with differential gene expression (**b**), the numbers of treatment positives are also shown, as empty black down triangles. In each panel, treatments are ordered according to the number of DEGs identified with SVCD normalization, increasing from left to right (see Supplementary Table 2, for real dataset). Differential gene expression was analyzed with R/Bioconductor package limma. Supplementary Fig. S2 shows results obtained with *t*-tests, qualitatively similar but with much lower detection of differential gene expression.

Figure 3 shows the numbers of DEGs detected in the real and synthetic datasets, for each of the 42 experimental treatments compared to the corresponding control (Supplementary Table S2), after normalizing with the four methods. For the real dataset (Fig. 3a), the number of DEGs identified after MedianCD and SVCD normalization were much larger for most treatments, in some cases by more than one order of magnitude. For the synthetic dataset with differential gene expression (Fig. 3b), results were qualitatively similar, but with less differential gene expression detected, consistently with Fig. 2a,b. The number of treatment positives can be displayed in this case (empty black down triangles, Fig. 3b), showing a better correlation, with MedianCD and SVCD normalization, between the number of treatment positives and detected positives. For the synthetic dataset without differential gene expression (Fig. 3c), no DEG was found but for one or two DEGs in two conditions. Given that the false discovery rate was controlled to be less than 5% per treatment, this is expected to happen when evaluating 42 treatments.

**Figure 4:**
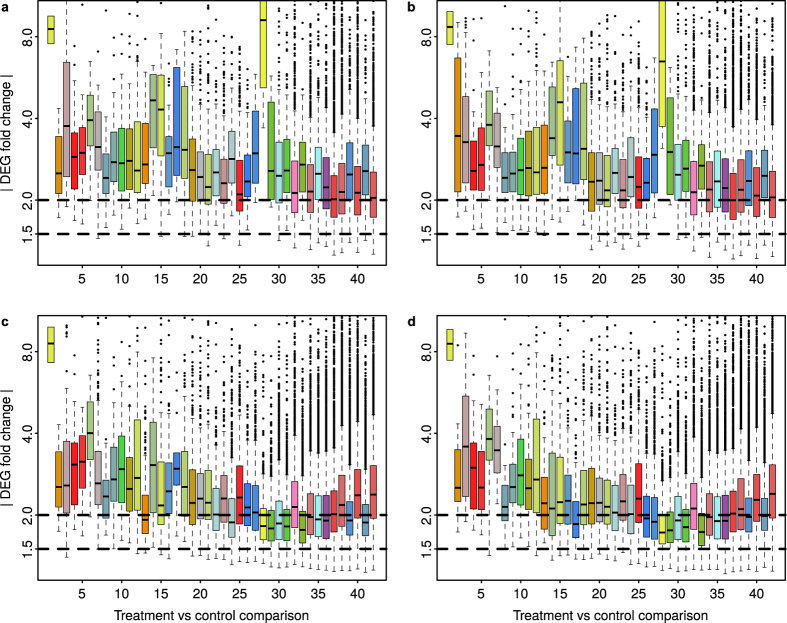
For the real dataset, MedianCD and SVCD normalization allowed to detect variation in gene expression of smaller magnitude than with Median and Quantile normalization. Boxplots display absolute values of DEG fold changes, for each treatment compared to the corresponding control, obtained after Median normalization (**a**), Quantile normalization (**b**), MedianCD normalization (**c**), and SVCD normalization (**d**). Boxplots are colored by treatment, with the same color code as in Fig. 1. All panels have the same order of treatments as in Fig. 3a, i.e. in increasing number of DEGs identified with SVCD normalization (Supplementary Table 2). Dashed horizontal lines indicate references of 1.5-fold and 2-fold changes.

Figure 3a reports, for the real dataset, statistically significant changes of gene expression, that is, changes that cannot be explained by chance. Equally important is the effect size, i.e. the scale of detected variation in DEGs, which is displayed by Fig. 4. The boxplots show absolute fold changes of expression level for all DEGs detected after applying each normalization method. MedianCD and SVCD normalization allowed to detect smaller changes of gene expression, which were otherwise missed when using Median and Quantile normalization. This differential gene expression detected with MedianCD and SVCD normalization can hardly be considered negligible, given that, for all treatments, the interquartile range of absolute fold changes was above 1.5-fold, and, for more than 28 (67%) treatments, the median absolute fold change was greater than 2-fold. Interestingly, the scale of differential gene expression detected with MedianCD and SVCD normalization in this assay is of similar magnitude to those reported by studies of global mRNA changes using external controls with microarrays and/or RNA-Seq^37^,^39^.

**Figure 5:**
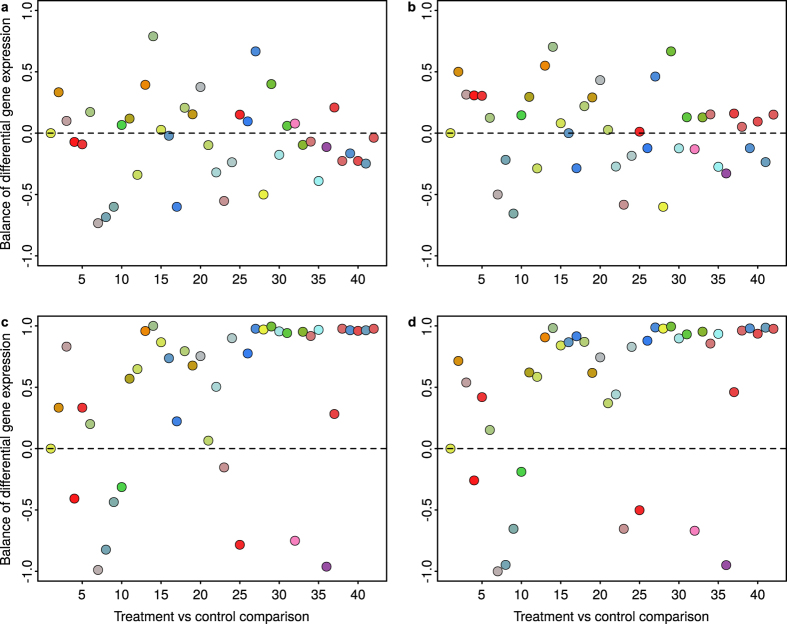
For the real dataset, detected differential gene expression was unbalanced, specially after using MedianCD and SVCD normalization and for the treatments with more DEGs (Fig. 3a). Panels show the balance of differential gene expression, for each treatment compared to the corresponding control, obtained after Median normalization (**a**), Quantile normalization (**b**), MedianCD normalization (**c**), and SVCD normalization (**d**). Each point represents the balance of differential gene expression, 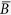 (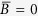, same number of over- and under-expressed genes; 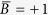, all DEGs over-expressed; 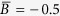, 75% DEGs under-expressed). Points are colored by treatment, with the same color code as in Figs 1 and 4. All panels have the same order of treatments as in Figs 3a and 4, i.e. in increasing number of DEGs identified with SVCD normalization (Supplementary Table 2). Dashed horizontal lines indicate references for balanced differential gene expression (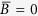).

Figure 5 displays the balance of differential gene expression, i.e. the comparison between the number of over- and under-expressed genes, for the real dataset. The quantity in the *y*-axes is the mean of an indicator variable *B*, which assigns +1 to each over-expressed DEG and −1 to each under-expressed DEG. Hence, balance of differential gene expression corresponds to 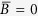, all DEGs over-expressed to 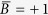, and, for example, 60% DEGs under-expressed to 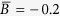. As discussed below, and as it has been reported before^45^,^46^,^51^,^52^, the balance of differential gene expression has a strong impact on the performance of normalization methods. Figure 5 shows that, regardless of the normalization method used, the unbalance of differential gene expression detected in the real dataset was substantial for most conditions. Detected unbalances were (in absolute value) larger with MedianCD and SVCD normalization, in both cases with more than 30 (71%) treatments having 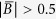, that is, more than 75% of over- or under-expressed genes. Moreover, the differences between the unbalances detected with Median and Quantile normalization, on one hand, and MedianCD and SVCD normalization, on the other, were specially notorious for the treatments with more DEGs (treatments 26–42, Fig. 3a). In those cases, Median and Quantile normalization resulted in the smallest detected unbalances, whereas MedianCD and SVCD normalization yielded the largest ones, with values near 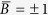 for all but two treatments.

**Figure 6:**
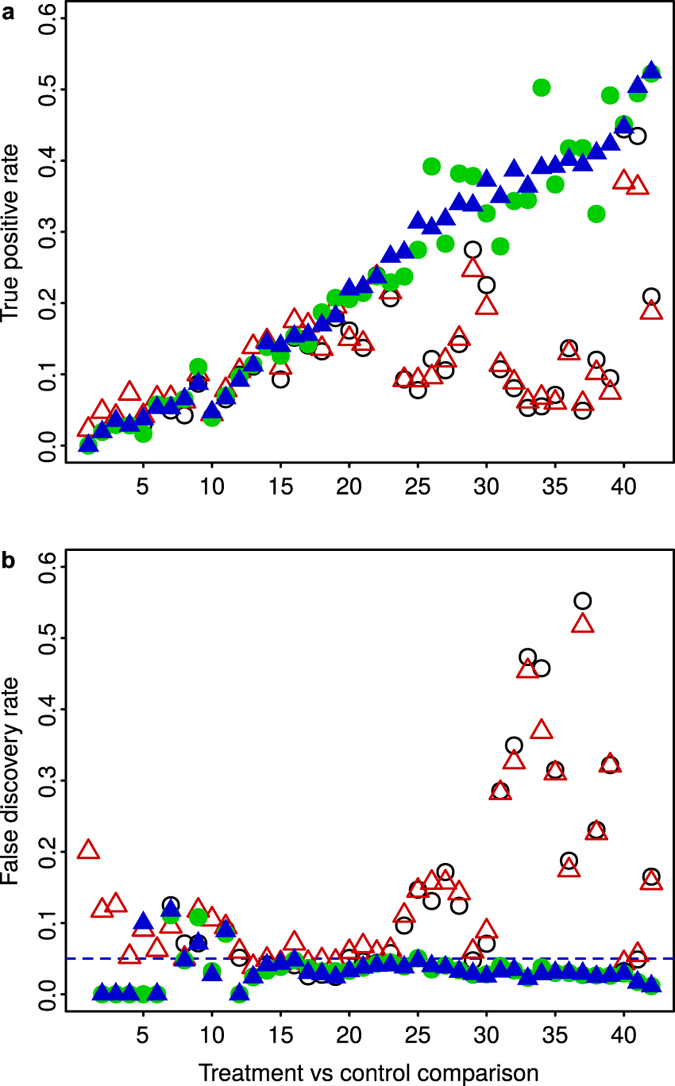
In the synthetic dataset with differential gene expression, and with more than 10% of treatment positives (Fig. 3b), Median and Quantile normalization resulted in less statistical power and uncontrolled false discovery rate. The panels display the true positive rate (**a**) and false discovery rate (**b**), for each treatment compared to the corresponding control, obtained after applying the four normalization methods (same symbols as in Fig. 3; empty black circles, Median normalization; empty red up triangles, Quantile normalization; filled green circles, MedianCD normalization; filled blue up triangles, SVCD normalization). Both panels have the same order of treatments as in Fig. 3b, i.e. in increasing number of differentially expressed genes identified with SVCD normalization. Differential gene expression was analyzed with R/Bioconductor package limma. The dashed horizontal line in (**b**) indicates the desired bound on the false discovery rate at 0.05.

True differential expression was known, by construction, for the synthetic dataset with differential gene expression. Thus, Fig. 6 shows for this dataset the true positive rate (ratio between true positives and treatment positives, also known as statistical power or sensitivity) and the false discovery rate (FDR, ratio between false positives and detected positives). With conditions 1 to 20, which correspond to those conditions with less than approximately 10% of treatment positives (Fig. 3b, empty black down triangles), the true positive rate was similarly low for all normalizations. Regarding the FDR, when the (total) number of detected positives was up to a few tens, variability of the FDR around the target bound at 0.05 is to be expected, given that the bound is defined over an average of repetitions of the multiple-hypothesis test. Yet, the FDR obtained after Median and Quantile normalization was higher than the 0.05 bound for most conditions. More striking, however, was the behavior for conditions 21 to 42 (more than 10% of treatment positives). The true positive rates obtained after Median and Quantile normalization were much lower than those obtained with MedianCD and SVCD normalization, while the FDR after Median and Quantile normalization was clearly over the bound at 0.05. In comparison, MedianCD and SVCD normalization, besides offering better sensitivity of differential gene expression, maintained the FDR consistently below the desired bound.

**Figure 7:**
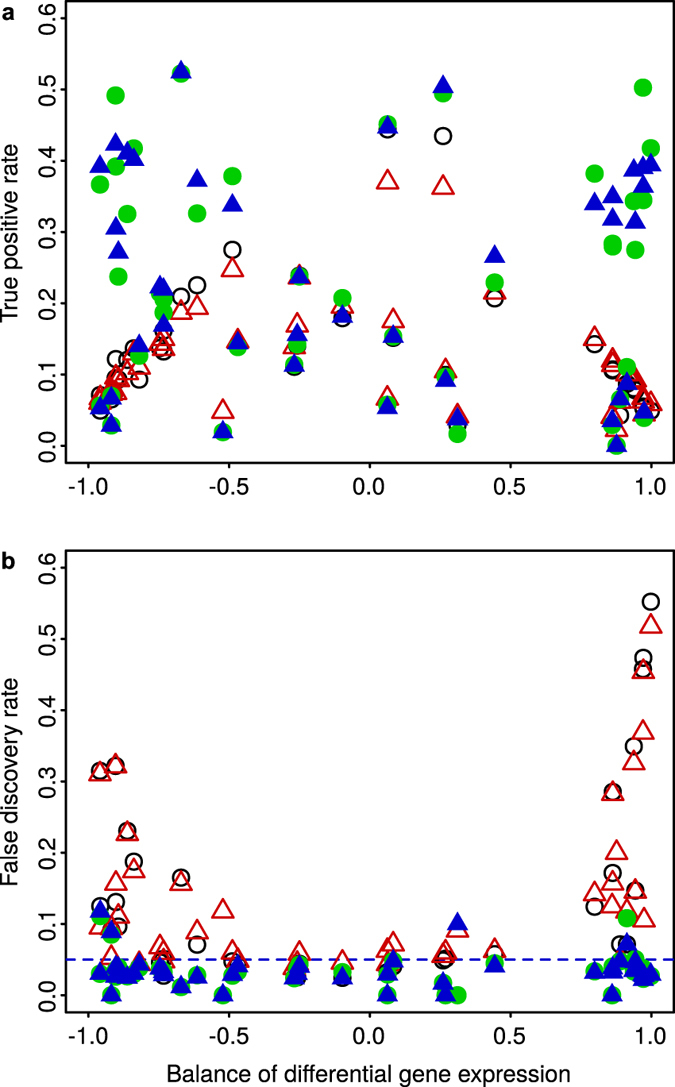
In the synthetic dataset with differential gene expression (Fig. 6), the unbalance between over- and under-expressed genes was a key factor in the lowered true positive rate and uncontrolled false discovery rate obtained after Median and Quantile normalization. Panels show the true positive rate (**a**) and false discovery rate (**b**) as a function of the balance of differential gene expression, 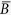 (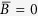, same number of over- and under-expressed genes; 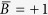, all DEGs over-expressed; 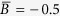, 75% DEGs under-expressed). Each point in both panels represents the results for one treatment compared to the corresponding control, obtained after applying the four normalization methods (same symbols as in Figs 3 and 6; empty black circles, Median normalization; empty red up triangles, Quantile normalization; filled green circles, MedianCD normalization; filled blue up triangles, SVCD normalization). Differential gene expression was analyzed with R/Bioconductor package limma. The dashed horizontal line in (**b**) indicates the desired bound on the false discovery rate at 0.05.

Figure 7 further explores these results, by representing the true positive rate and false discovery rate (FDR) as a function of the unbalance between over- and under-expressed genes. Figure 7 shows that the unbalance of differential gene expression was a key factor in the results obtained with Median and Quantile normalization. When most DEGs were over- or under-expressed, both the true positive rate and FDR degraded markedly after using Median or Quantile normalization. In contrast, the true positive rate and FDR were not affected by the unbalance of differential gene expression when using MedianCD or SVCD normalization.

Concerning the identification of no-variation genes, both MedianCD and SVCD normalization performed well. In the synthetic dataset without differential gene expression, both methods identified all genes as no-variation genes, which is the best possible result. In the synthetic dataset with differential gene expression, 1,834 genes (10% of a total of 18,339 genes) were, by construction, negatives across all treatments. MedianCD and SVCD normalization detected, respectively, 1,723 and 1,827 no-variation genes, among which 96.9% and 95.2% were true negatives.

### Analysis of the Golden Spike and Platinum Spike Datasets

To provide additional evidence of the performance of MedianCD and SVCD normalization, we analyzed the Golden Spike^53^ and Platinum Spike^52^ datasets. Both of them are artificial real datasets, the largest ones for which true DEGs are known. Hence, they have been widely used to benchmark normalization methods^45^,^46^,^47^,^52^,^53^,^54^,^55^.

The design of the Golden Spike dataset was questioned for reasons concerning, among others, the anomalous null distribution of *p*-values, the lack of biological replicates, and the high concentration of spike-ins^51^,^56^,^57^. Nevertheless, this dataset is worth considering here because it challenges what we claim are key capabilities of our approach, that is, to correctly normalize gene expression data when many genes are differentially expressed, even with large unbalance between over- and under-expression. This dataset consists of microarray data obtained with the Affymetrix GeneChip DrosGenome1, with two experimental conditions and three technical replicates per condition. Excluding Affymetrix internal control probes, the dataset contains a total of 13,966 gene probe sets, of which 3,876 were spiked-in, which we call *known* in what follows. Among these, 1,328 (34.3%) were over-expressed (known positives) to varying degrees between 1.1- and 4-fold, while the remaining 2,535 (65.4%) were spiked-in at the same concentration in both conditions (known negatives). (Percentages do not add up to 100% because of a very small number of probe sets with weak matching to multiple clones^53^).

**Figure 8:**
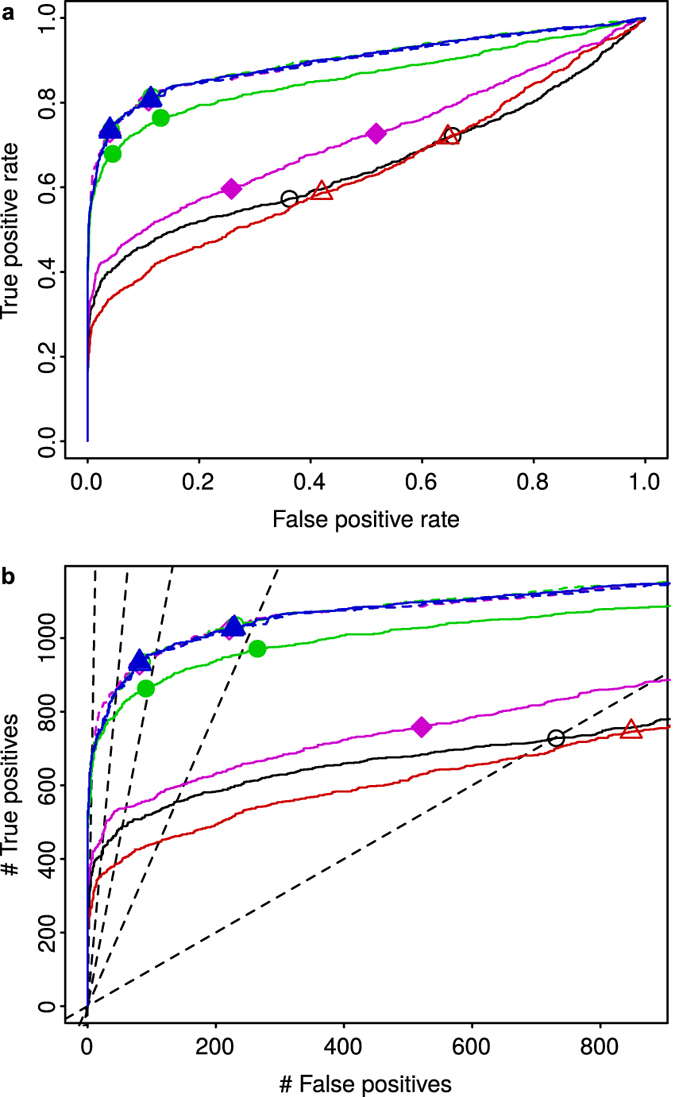
In the Golden Spike dataset, the best detection of differential gene expression was achieved after using MedianCD and, specially, SVCD normalization. Panels display ROC curves, with the true positive rate versus the false positive rate (**a**), or the number of true positives versus the number of false positives (**b**). Each curve shows the results obtained after applying the four normalization methods plus Cyclic Loess normalization (same colors and symbols as in Figs 3, 6 and 7; black curve with empty black circles, Median normalization; red curve with empty red up triangles, Quantile normalization; green curve with filled green circles, MedianCD normalization; blue curve with filled blue up triangles, SVCD normalization; magenta curve with filled magenta diamonds, Cyclic Loess normalization). Dashed curves with lightly filled symbols, overlapping the response of SVCD normalization, show results when the list of known negatives was provided to MedianCD, SVCD, and Cyclic Loess normalization. The two points per normalization method show results when controlling the false discovery rate (FDR) to be below 0.01 (left point) or 0.05 (right point). Dashed lines in (**b**) show references for actual FDR equal to 0.01, 0.05, 0.1, 0.2, or 0.5 (from left to right). Compared to MedianCD and SVCD normalization, the other normalization methods resulted in notably more severe degradation of the FDR.

In addition to the normalization methods used above, we included Cyclic Loess normalization^49^,^58^ in this case, because it facilitates a better comparison of results with previous studies^45^,^46^,^47^,^52^,^53^,^54^,^55^. Figure 8 summarizes the results obtained for the Golden Spike dataset, by displaying Receiver Operating Characteristic (ROC) curves for the detection of differential gene expression. The upper panel shows the true positive rate (as before, ratio between true positives and treatment positives) versus the false positive rate (ratio between false positives and treatment negatives), while the lower panel shows the number of true positives versus the number of false positives. In both cases, detected and treatment positives/negatives were restricted to known genes, following previous studies^54^,^55^,^57^. Doing otherwise would have given an excessively dominant role to the issue of cross-hybridization in the analysis of differential gene expression^54^. Additionally, the analysis was performed using only probe sets with hybridization signal in all samples, with the aim of factoring out differences between normalization methods caused by the response to missing data. Results obtained without this restriction (Supplementary Fig. S3) or with *t*-tests instead of limma^59^ analysis (Supplementary Fig. S4) were very similar to those of Fig. 8.

The comparison of ROC curves shown in Fig. 8 highlight the superior performance of MedianCD and, in particular, SVCD normalization. Dashed lines show results when the list of known negatives was given as an input to some of the normalization methods (something than cannot be done in real assays). It is remarkable that SVCD normalization featured equally well with or without this information.

Points in Fig. 8 indicate the results when controlling the false discovery rate (FDR) to be below 0.01 (left point on each curve) or 0.05 (right point). Figure 8b shows reference lines for actual FDR equal to 0.01, 0.05, 0.1, 0.2 and 0.5 (from left to right). In all cases, the FDR was not adequately controlled, although the difference between intended and actual FDR was notably smaller with MedianCD and SVCD normalization. Lack of control of the FDR in the analysis of this dataset has been previously reported^53^,^55^. It is caused by the non-uniform (hence anomalous) distribution of *p*-values for negative genes, which results from the analysis of differential gene expression^55^,^56^,^57^,^60^. It has been argued that this anomalous distribution of *p*-values is, in turn, a consequence of the own experimental design of the dataset, in particular the lack of biological replication and the way clone aliquots were mixed to produce each gene group with a given fold change^56^. Later studies have attributed this issue mostly to non-linear or intensity-dependent effects, not properly corrected in the within-sample normalization step (e.g. background correction) of the analysis pipeline^52^,^55^,^57^,^60^.

Concerning the identification of no-variation genes, both MedianCD and SVCD normalization worked correctly. MedianCD normalization identified 561 no-variation genes, of which 93.9% were known, and among which 84.1% were known negatives. SVCD normalization, in comparison, featured better detection, with 1,224 no-variation genes identified, of which 94.4% were known, and among which 90.0% were known negatives.

The design of the Platinum Spike dataset^52^ took into account the concerns raised by the Golden Spike dataset, offering a dataset with two experimental conditions and nine (three biological × three technical) replicates per condition, and including near 50% more spike-ins. Besides, differential gene expression was balanced, with respect to both total mRNA amount and extent of over- and under-expression. Gene expression data was obtained with Affymetrix Drosophila Genome 2.0 microarrays. Excluding Affymetrix internal control probes, the dataset contained a total of 18,769 probe sets, of which 5,587 were spiked-in, called *known* as above. Among these, 1,940 (34.7%) were differentially expressed (known positives) to varying degrees between 1.2- and 4-fold (1,057 over-expressed, 883 under-expressed), while the remaining 3,406 (61.0%) were spiked-in at the same concentration in both conditions (known negatives).

**Figure 9:**
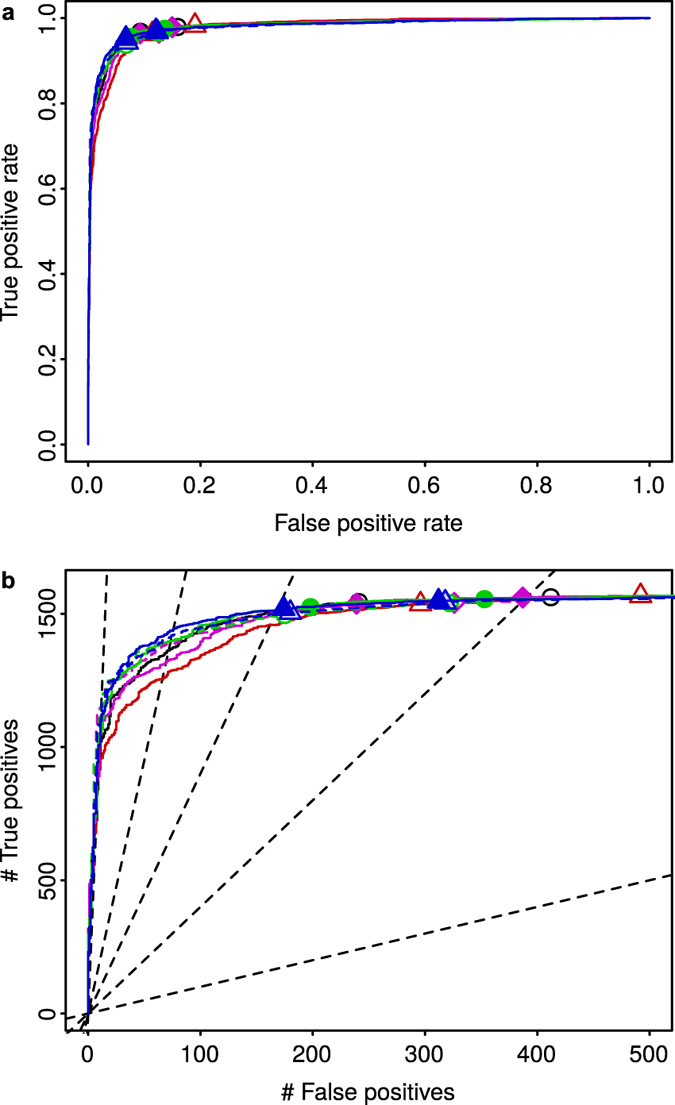
In the Platinum Spike dataset, all normalization methods resulted in similar detection of differential gene expression, with MedianCD and SVCD normalization being only marginally better. Panels display ROC curves, with the true positive rate versus the false positive rate (**a**), or the number of true positives versus the number of false positives (**b**). Each curve shows the results obtained after applying the four normalization methods plus Cyclic Loess normalization (same colors and symbols as in Figs 3, 6, 7 and 8; black curve with empty black circles, Median normalization; red curve with empty red up triangles, Quantile normalization; green curve with filled green circles, MedianCD normalization; blue curve with filled blue up triangles, SVCD normalization; magenta curve with filled magenta diamonds, Cyclic Loess normalization). Dashed curves with lightly filled symbols show results when the list of known negatives was provided to MedianCD, SVCD, and Cyclic Loess normalization. As in Fig. 8, the two points per normalization method show results when controlling the false discovery rate (FDR) to be below 0.01 (left point) or 0.05 (right point). Dashed lines in (**b**) show references for actual FDR equal to 0.01, 0.05, 0.1, 0.2, or 0.5 (from left to right). Compared to the Golden Spike dataset (Fig. 8), the difference between normalization methods in the resulting degradation of the FDR was smaller for this dataset.

Figure 9 shows ROC curves for the Platinum Spike dataset. As above, only known genes were considered for detected and treatment positives/negatives. Additionally, gene probes were restricted to those with signal in all samples. Results obtained without this restriction (Supplementary Fig. S5) or with *t*-tests instead of limma analysis (Supplementary Fig. S6) were again very similar. In contrast to the Golden Spike dataset (Fig. 8), the performance concerning true and false positives resulting from the different normalization methods was much more comparable. In this case, MedianCD and SVCD normalization were only marginally better. Note, however, that the FDR was again not properly controlled (Fig. 9b). Similarly to the Golden Spike dataset, and despite biological replication and a different experimental setup, obtained distributions of *p*-values for negative genes have been reported to be non-uniform^52^. This fact is consistent with previous arguments relating the lack of control of the FDR to a general problem concerning the correction of non-linearities in the preprocessing of microarray data^52^,^55^,^57^,^60^.

Regarding the identification of no-variation genes, MedianCD and SVCD normalization also worked correctly with this dataset. MedianCD normalization identified 2,090 no-variation genes, of which 95.4% were known, and among which 98.7% were known negatives. SVCD normalization featured slightly better, with 2,232 no-variation genes identified, of which 95.3% were known, and among which 98.3% were known negatives.

## Discussion

The lack-of-variation assumption underlying current methods of normalization was self-fulfilling, removing variation in gene expression that was actually present. Moreover, it had negative consequences for downstream analyses, as it removed potentially important biological information and introduced errors in the detection of gene expression. The resulting decrease in statistical power or sensitivity is a handicap, which can be addressed by increasing the number of samples per experimental condition. However, degradation of the (already weak) control of the false discovery rate when using Median or Quantile normalization is a major issue for real-world applications.

The removal of variation can be understood as additive errors in the estimation of normalization factors. Considering data and errors vectorially (see Methods), the length of each vector equals, after centering and up to a constant factor, the standard deviation of the data or error. Errors of small magnitude, compared to the data variance, would only have minor effects. However, errors of similar or greater magnitude than the data variance may, depending on the vector lengths and the angle between the vectors, severely distort the observed data variance. This will, in turn, cause spurious results in the statistical analyses. Furthermore, the angles between the data and the correct normalization factors (considered as vectors) are random, given that expression data reflect biological variation while normalization factors respond to technical variation. If the assay is repeated, even with exactly the same experimental setup, the errors in the normalization factors will vary randomly, causing random spurious results in the downstream analyses. This explains, at least partially, the lack of reproducibility found in transcriptomics studies, especially for the detection of changes in gene expression of small-to-medium magnitude (up to 2-fold), because variation of this size is more likely to be distorted by errors in the estimation of normalization factors. Accordingly, the largest differences in numbers of differentially expressed genes detected by Median and Quantile normalization, compared to MedianCD and SVCD normalization, occurred in the treatments with the smallest magnitudes of gene expression changes (Figs 3a and 4).

The variation between medians displayed in Fig. 1j,k,m,n may seem surprising, given routine expectations based on current methods (Fig. 1d,e,g,h). Nevertheless, this variation inevitably results from the unbalance between over- and under-expressed genes. As an illustration of this issue, let us consider a case with two experimental conditions, in which the average expression of a given gene is less than the distribution median under one condition, but greater than the median under the other. The variation of this gene alone will change the value of the median to the expression level of the next ranked gene. Therefore, if the number of over-expressed genes is different from the number of under-expressed genes, and enough changes cross the median boundary, then the median will substantially differ between conditions. Only when differential expression is negligible or is balanced with the respect to the median, will the median stay the same. Note that this is a related but different requirement from the number of over- and under-expressed genes being the same. This argument applies equally to any other quantile in the distribution of gene expression. The case of Quantile normalization is the least favorable, because it requires that changes of gene expression are balanced with respect to all distribution quantiles.

Compared with other normalization approaches that try to identify no-variation genes from expression data, such as Cross-Correlation^44^, LVS^45^, or NVAS^46^ normalization, our proposal is able to work correctly with higher degrees of variation in gene expression, given that those methods are not expected to work correctly when more than 50–60% of genes vary. The reason for this difference in performance lies in that those methods use a binning strategy over the average expression between conditions (Cross-Correlation, NVAS), or need to assume an a priori fraction (usually 40–60%) of non-differentially expressed genes (LVS). When the majority of genes are differentially expressed, very few of those bins may be suitable for normalization, or the assumed fraction of non-differentially expressed genes may not hold. In contrast, our approach makes one single search in a space of *p*-values, and without assuming any fraction of non-differentially expressed genes. As long as there are a sufficient number of non-differentially expressed genes, of the order of several hundreds, normalization is possible, including cases with global mRNA changes or transcriptional amplification^37^,^38^,^39^,^41^. In general, it is a matter of comparison between the magnitude of the error in the estimation of normalization factors and the amount of biological variation. The estimation error decreases with the number of no-variation genes detected (Fig. 2), and whenever normalization error is well below biological variation, normalization between samples will be correct and beneficial for downstream analyses.

Our approach to normalization is based in four key ideas: first, decomposing the normalization by experimental conditions and normalizing separately each condition before normalizing the condition means; second, using the novel Standard-Vector normalization (or alternatively median scaling) to perform each normalization; third, identifying no-variation genes from the distribution of *p*-values resulting from a statistical test of variation between conditions; and fourth, employing only no-variation genes for the final between-condition normalization. These four ideas are grounded on rigorous mathematical statistics (see Methods and Supplementary Information). It is also worth noting that both Median and Standard-Vector normalization, as methods for each normalization step, are distribution-free methods; they do not assume Gaussianity or any other kind of probability distribution for the expression levels of genes. MedianCD and SVCD normalization are freely available in the R package *cdnormbio*, installable from GitHub (see Code Availability).

Previous assumptions that gene variation is rather limited could suggest that there is no need for more comprehensive normalization methods such as our proposal. In line with this, it could be argued that the amount of variation in our real (*E. crypticus*) dataset is exceptional and much larger than the variation likely to be occur in most experiments. We think that this an invalid belief. Most of the available evidence concerning widespread variation in gene expression is inadequate, because it involves circular reasoning. We have shown here that current normalization methods, used by almost all studies to date, assume no variation in gene expression between experimental conditions, and they remove it if it exists, unless it is balanced. Therefore, these methods *cannot* be used to discern the extent and balance of global variation in gene expression. Only methods that are able to normalize correctly, whatever these extent and balance are, can be trusted for this task. The fact that our methods perform well with large and unbalanced differential gene expression does not imply that they perform poorly when differential gene expression is more moderate or balanced. Our results show that this is not case. In the design of our methods, no compromise was made to achieve good performance with high variation in exchange for not so good performance with low variation. The downside of our approach lies elsewhere, in a greater algorithmic complexity and a greater demand of computing resources. Yet, we consider this a minor demand, given the capabilities of today’s computers and the resources required by current high-throughput assays.

Our results have being obtained from microarray data, but similar effects are expected to be found in RNA-Seq assays. Current normalization procedures for RNA-Seq, such as RPKM^4^, TMM^35^, or DESeq^36^, perform between-sample normalization based on some form of global scaling and under the assumption that most genes are not differentially expressed. This makes RPKM, TMM, and DESeq normalization, in what concerns between-sample normalization and the removal or distortion of variation discussed here, similar to conventional Median normalization. An example of this issue, including results from microarray and RNA-Seq assays, has been reported in a study of the transcriptional amplification mediated by the oncogene c-Myc^39^.

Importantly, MedianCD and SVCD normalization were designed with no dependencies on any particular aspect of the technology used to globally measure gene expression, i.e. microarrays or RNA-Seq. The numbers in the input data are interpreted as steady state concentrations of mRNA molecules, in order to identify the normalization factors, and irrespectively of whether the concentrations were obtained from fluorescence intensities of hybridized cDNA (microarrays) or from counts of fragments of cDNA (RNA-Seq). Both technologies require between-sample normalization, because in some step of the assay the total mRNA or cDNA mass in each sample must be equalized within a given range required by the experimental platform, This equalization of total mass, together with other sources of variation in the total efficiency of the assay, amounts to a factor multiplying the concentration of each mRNA species. This factor is different for each sample, and it is what between-sample normalization aims to detect and correct for. Moreover, the total mRNA mass in each sample is, in many cases, mostly determined by a few highly expressed genes, rather than an unbiased average over the total mRNA population. This makes between-sample normalization critical regarding comparisons of gene expression between different experimental conditions, as our results illustrate. It is also important to highlight that this between-sample uncertainty in the measurement of mRNA concentrations is different from other issues, such as for example non-linearities. These other problems are usually more specific to each technology, and they are the scope of within-sample normalization (e.g. background correction for microarrays and gene-length normalization for RNA-Seq), which are obviously also necessary and should be applied *before* between-sample normalization. Similarly, methods that address the influence of biological or technical confounding factors on downstream analyses, such as SVA^61^ or PEER^62^, should be applied when necessary, *after* normalizing.

Finally, the significance of widespread variation in gene expression merits consideration from the viewpoint of molecular and cell biology. Established understanding about the regulation of gene expression considers it as a set of processes that generally switch on or off the expression of genes, performed mostly at transcription initiation, by the combinatorial regulation of a large number of transcription factors, and with an emphasis on gene expression programs associated with cell differentiation and development. Recent studies, however, have expanded this understanding, offering a more complex perspective on the regulation of gene expression, by identifying other rate-limiting regulation points between transcription initiation and protein translation, such as transcription elongation and termination, as well as mRNA processing, transport and degradation. Promoter-proximal pausing of RNA polymerase II (in eukaryotes)^63^,^64^ and transcript elongation^65^, in particular, have received a great deal of attention recently, in connection with gene products involved in signal transduction pathways. These mechanisms, which seem to be highly conserved among metazoans, would allow cells to tune the expression of activated genes in response to signals concerning, for example, homeostasis, environmental stress or immune response. As an illustration, studies about the transcription amplification mediated by the oncogene c-Myc have uncovered that it regulates the promoter-proximal pausing of RNA polymerase II, affecting a large number of genes already activated by other regulatory mechanisms^66^,^67^,^68^. Our results for the toxicity experiment with *E. crypticus* are consistent with regulatory capabilities for broad fine-tuning of gene expression levels, far beyond what conventional methods of normalization would allow to detect. This contrast underlines that normalization methods that truly preserve variation between experimental conditions are necessary for high-throughput assays exploring genome-wide regulation of gene expression, as required by current research in molecular and cell biology.

In summary, this study proves that large numbers of genes can change in expression level across experimental conditions, and too extensively to ignore in the normalization of gene expression data. Current normalization methods for gene expression microarrays and RNA-Seq, because of a lack-of-variation assumption, likely remove and distort variation in gene expression. The normalization methods proposed here solve this problem, offering a means to investigate broad changes in gene expression that have remained hidden to date. We expect this to provide revealing insights about diverse biomolecular processes, particularly those involving substantial numbers of genes, and to assist efforts to realize the full potential of gene expression profiling.

## Methods

### Test Organism and Exposure Media

The test species was *Enchytraeus crypticus*. Individuals were cultured in Petri dishes containing agar medium, in controlled conditions^69^.

For copper (Cu) exposure, a natural soil collected at Hygum, Jutland, Denmark was used^69^,^70^. For silver (Ag) and nickel (Ni) exposure, the natural standard soil LUFA 2.2 (LUFA Speyer, Germany) was used^69^. The exposure to ultra-violet (UV) radiation was done in ISO reconstituted water^71^.

### Test Chemicals

The tested Cu forms^69^ included copper nitrate (Cu(NO_3_)_2_ · 3H_2_O > 99%, Sigma Aldrich), Cu nanoparticles (Cu-NPs, 20–30 nm, American Elements) and Cu nanowires (Cu-Nwires, as synthesized^72^).

The tested Ag forms^69^ included silver nitratre (AgNO_3_ > 99%, Sigma Aldrich), non-coated Ag nanoparticles (Ag-NPs Non-Coated, 20–30 nm, American Elements), Polyvinylpyrrolidone (PVP)-coated Ag nanoparticles (Ag-NPs PVP-Coated, 20–30 nm, American Elements), and Ag NM300K nanoparticles (Ag NM300K, 15 nm, JRC Repository). The Ag NM300K was dispersed in 4% Polyoxyethylene Glycerol Triolaete and Polyoxyethylene (20) orbitan mono-Laurat (Tween 20), thus the dispersant was tested alone as control (CTdisp).

The tested Ni forms included nickel nitrate (Ni(NO_3_)_2_ · 6H_2_O ≥ 98.5%, Fluka) and Ni nanoparticles (Ni-NPs, 20 nm, American Elements).

### Spiking Procedure

Spiking for the Cu and Ag materials was done as previously described^69^. For the Ni materials, the Ni-NPs were added to the soil as powder, following the same procedure as for the Cu materials. NiNO_3_, being soluble, was added to the pre-moistened soil as aqueous dispersions.

The concentrations tested were selected based on the reproduction effect concentrations EC_20_ and EC_50_, for *E. crypticus*, within 95% of confidence intervals, being: CuNO_3_ EC_20/50_ = 290/360 mgCu/kg, Cu-NPs EC_20/50_ = 980/1760 mgCu/kg, Cu-Nwires EC_20/50_ = 850/1610 mgCu/kg, Cu-Field EC_20/50_ = 500/1400 mgCu/kg, AgNO_3_ EC_20/50_ = 45/60 mgAg/kg, Ag-NP PVP-coated EC_20/50_ = 380/550 mgAg/kg, Ag-NP Non-coated EC_20/50_ = 380/430 mgAg/kg, Ag NM300K EC_20/50_ = 60/170 mgAg/kg, CTdisp = 4% w/w Tween 20, NiNO_3_ EC_20/50_ = 40/60 mgNi/kg, Ni-NPs EC_20/50_ = 980/1760 mgNi/kg.

Four biological replicates were performed per test condition, including controls. For Cu exposure, the control condition for all the treatments consisted of soil from a control area at Hygum site, which has a Cu background concentration of 15 mg/kg^70^. For Ag exposure, two control sets were performed: CT (un-spiked LUFA soil, to be the control condition for AgNO_3_, Ag-NPs PVP-Coated and Ag-NPs Non-Coated treatments) and CTdisp (LUFA soil spiked with the dispersant Tween 20, to be the control condition for the Ag NM300K treatments). For Ni exposure, the control consisted of un-spiked LUFA soil.

### Exposure Details

In soil (i.e. for Cu, Ag and Ni) exposure followed the standard ERT^73^ with adaptations as follows: twenty adults with well-developed clitellum were introduced in each test vessel, containing 20 g of moist soil (control or spiked). The organisms were exposed for three and seven days under controlled conditions of photoperiod (16:8 h light:dark) and temperature 20 ± 1°C without food. After the exposure period, the organisms were carefully removed from the soil, rinsed in deionized water and frozen in liquid nitrogen. The samples were stored at −80 °C, until analysis.

For UV exposure, the test conditions^71^ were adapted for *E. crypticus*^74^. The exposure was performed in 24-well plates, where each well corresponded to a replicate and contained 1 ml of ISO water and five adult organisms with clitellum. The test duration was five days, at 20 ± 1°C. The organisms were exposed to UV on a daily basis, during 15 minutes per day to two UV intensities (280–400 nm) of 1669.25 ± 50.83 and 1804.08 ± 43.10 mW/m^2^, corresponding to total UV doses of 7511.6 and 8118.35 J/m^2^, respectively. The remaining time was spent under standard laboratory illumination (16:8 h photoperiod). UV radiation was provided by an UV lamp (Spectroline XX15F/B, Spectronics Corporation, NY, USA, peak emission at 312 nm) and a cellulose acetate sheet was coupled to the lamp to cut-off UVC-range wavelengths^74^. Thirty two replicates per test condition (including control without UV radiation) were performed to obtain 4 biological replicates for RNA extraction, each one with 40 organisms. After the exposure period, the organisms were carefully removed from the water and frozen in liquid nitrogen. The samples were stored at −80 °C, until analysis.

### RNA Extraction, Labeling and Hybridization

RNA was extracted from each replicate, which contained a pool of 20 and 40 organisms, for soil and water exposure, respectively. Three biological replicates per test treatment (including controls) were used. Total RNA was extracted using SV Total RNA Isolation System (Promega). The quantity and purity were measured spectrophotometrically with a nanodrop (NanoDrop ND-1000 Spectrophotometer) and its quality checked by denaturing formaldehyde agarose gel electrophoresis.

500 ng of total RNA were amplified and labeled with Agilent Low Input Quick Amp Labeling Kit (Agilent Technologies, Palo Alto, CA, USA). Positive controls were added with the Agilent one-color RNA Spike-In Kit. Purification of the amplified and labeled cRNA was performed with RNeasy columns (Qiagen, Valencia, CA, USA).

The cRNA samples were hybridized on custom Gene Expression Agilent Microarrays (4 × 44k format), with a single-color design^48^. Hybridizations were performed using the Agilent Gene Expression Hybridization Kit and each biological replicate was individually hybridized on one array. The arrays were hybridized at 65°C with a rotation of 10 rpm, during 17 h. Afterwards, microarrays were washed using Agilent Gene Expression Wash Buffer Kit and scanned with the Agilent DNA microarray scanner G2505B.

### Data Acquisition

Fluorescence intensity data was obtained with Agilent Feature Extraction Software v. 10.7.3.1, using recommended protocol GE1_107_Sep09. Quality control was done by inspecting the reports on the Agilent Spike-in control probes.

### Data Analysis

Analyses were performed with R^75^ v. 3.3.1, using R packages plotrix^76^ v. 3.6.3 and RColorBrewer^77^ v. 1.1.2, and with Bioconductor^78^ v. 3.3 packages affy^79^ v. 1.50.0, drosgenome1.db v.3.2.3, drosophila2.db v. 3.2.3, genefilter v. 1.54.2, and limma^59^ v. 3.28.20. Background correction was carried out by Agilent Feature Extraction software for the real (*E. crypticus*) dataset, while the Affymetrix MAS5 algorithm, as implemented in the limma package, was used for the Golden and Platinum Spike datasets.

To ensure an optimal comparison between the different normalization methods, only gene probes with good signal quality (flag IsPosAndSignif = True) in all samples were employed for the analysis of the *E. crypticus* dataset. This implied the selection of 18,339 gene probes from a total of 43,750. For the Golden and Platinum Spike datasets, data were considered as missing when probe sets were not called present by the MAS5 algorithm.

The synthetic dataset without differential gene expression was generated gene by gene as normal variates with mean and variance equal, respectively, to the sample mean and sample variance of the expression levels for each gene, as detected from the real *E. crypticus* dataset after SVCD normalization. The synthetic dataset with differential gene expression was generated equally, except for the introduction of differences in expression averages between treatments and controls. The magnitude of the difference in averages was equal, for each differentially expressed gene (DEG), to twice the sample variance. The percentage of DEGs for each treatment was chosen randomly, in logarithmic scale, from a range between 0.9% and 90%, while ensuring that 10% of genes were not differentially expressed across the entire dataset. One third of the treatments were mostly over-expressed (for each treatment independently, the probability of a DEG being over-expressed was 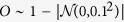), one third of the treatments were mostly under-expressed (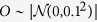), and the remaining third had mostly balanced differential gene expression 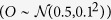. For both synthetic datasets, the applied normalization factors were those detected by SVCD normalization from the real *E. crypticus* dataset.

Median normalization was performed, for each sample, by subtracting the median of the distribution of expression levels, and then adding the overall median to preserve the global expression level. Quantile normalization was performed as implemented in the limma package.

The two condition-decomposition normalizations, MedianCD and SVCD, proceeded in the same way: first, independent within-condition normalization for each experimental condition, using all genes. Then, one between-condition normalization, iteratively identifying no-variation genes and normalizing until convergence of the set of no-variation genes. And finally, another between-condition normalization using only the no-variation genes detected, to calculate the between-condition normalization factors.

The criterion for convergence of MedianCD normalization was to require that the relative changes in the standard deviation of the normalization factors were less than 0.1%, or less than 10% for 10 steps in a row. In the case of SVCD normalization, convergence required that numerical errors were, compared to estimated statistical errors (see below), less than 1%, or less than 10% for 10 steps in a row. Convergence of the set of no-variation genes was achieved by intersection of the sets found during 10 additional steps under convergence conditions. These default convergence parameters were used for all the MedianCD and SVCD normalizations reported, with the exception of MedianCD with the Golden Spike dataset, which used 30% (instead of 10%) of relative change for 10 steps in a row, to reach convergence.

In SVCD normalization, the distribution of standard vectors was trimmed in each step to remove the 1% more extreme values of variance.

Differentially expressed genes were identified with limma analysis or t-tests, controlling the false discovery rate to be below 5%, independently for each comparison of treatment versus control.

The reference distributions with permutation symmetry shown in the polar plots of Supplementary Movies S1–S3 were calculated through the six possible permutations of the empirical standard vectors. The Watson *U*^2^ statistic was calculated with the two-sample test^80^, comparing with an equal number of samples obtained by sampling with replacement the permuted standard vectors.

### Condition Decomposition of the Normalization Problem

In a gene expression dataset with *g* genes, *c* experimental conditions and *n* samples per condition, the *observed* expression levels of gene *j* in condition *k*, 
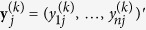
, can be expressed in log_2_-scale as


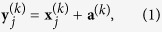


where 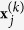 is the vector of *true* gene expression levels and ***a***^(*k*)^ is the vector of normalization factors.

Given a sample vector ***x***, the mean vector is 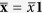, and the residual vector is 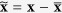. Then, (1) can be linearly decomposed into


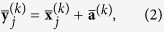



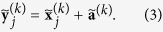


Equations (3) define the within-condition normalizations for each condition *k*. The scalar values in (2) are used to obtain the equations on condition means,


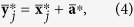



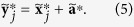


The between-condition normalization is defined by (5). Equations (4) reduce to a single number, which is irrelevant to the normalization. The complete solution for each condition is obtained with 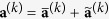.

For full details about this condition-decomposition approach, see Supplementary Mathematical Methods in the Supplementary Information.

### Standard-Vector Normalization

The *n* samples of gene *j* in a given condition can be modeled with the random vectors 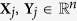. Again, ***Y***_*j*_ = ***X***_*j*_ + ***a***, where ***a*** is a fixed vector of normalization factors. It can be proved under fairly general assumptions (see Supplementary Information), that the true standard vectors have zero expected value


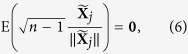


whereas the observed standard vectors verify, as long as **a** ≠ **0**,


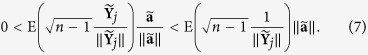


This motivates the following iterative procedure to solve (3) and (5) (*standard-vector normalization*):


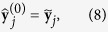



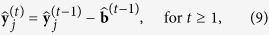



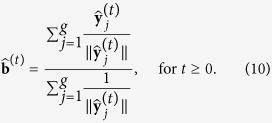


At convergence, 
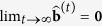
, which implies 
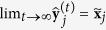
 and 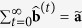. Convergence is faster the more symmetric the empirical distribution of 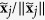 is on the unit (*n* − 2)-sphere. Convergence is optimal with spherically symmetric distributions, such as the Gaussian distribution, because in that case


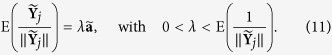


Assuming no dependencies between genes, an approximation of the statistical error at step *t* can be obtained with


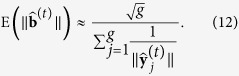


This statistical error was compared with the numerical error to assess convergence.

See Supplementary Mathematical Methods in the Supplementary Information for full details about this algorithm. See also Supplementary Movies S1–S3 for normalization examples.

### Identification of No-Variation Genes

No-variation genes were identified with one-sided Kolmogorov-Smirnov tests, as goodness-of-fit tests against the uniform distribution, carried out on a distribution of *p*-values. These *p*-values were obtained from ANOVA tests on the expression levels of genes, grouped by experimental condition. The KS test was rejected at *α* = 0.001.

See Supplementary Mathematical Methods in the Supplementary Information for more details about this approach to identify no-variation genes. See also Supplementary Movies S4–S6 for examples of use.

### Data Availability

MIAME-compliant microarray data were submitted to the Gene Expression Omnibus (GEO) repository at the NCBI website (platform: GPL20310; series: GSE69746, GSE69792, GSE69793 and GSE69794). The novel normalization methods were implemented as R functions. This code, together with R scripts that generate the synthetic datasets and reproduce all reported results starting from the raw microarray data, are available at the GitHub repository https://github.com/carlosproca/geneexpr-norm-paper and in the Supplementary Data File gene-expr-norm.zip. Additionally, MedianCD and SVCD normalization are available via the R package cdnormbio, installable from the GitHub repository https://github.com/carlosproca/cdnormbio.

## Additional Information

**How to cite this article:** Roca, C. P. *et al*. Variation-preserving normalization unveils blind spots in gene expression profiling. *Sci. Rep.* **7**, 42460; doi: 10.1038/srep42460 (2017).

**Publisher's note:** Springer Nature remains neutral with regard to jurisdictional claims in published maps and institutional affiliations.

## Supplementary Material

Supplementary Information

Supplementary Movie S1

Supplementary Movie S2

Supplementary Movie S3

Supplementary Movie S4

Supplementary Movie S5

Supplementary Movie S6

Supplementary Dataset 1
