# Corrigendum: Variation-preserving normalization unveils blind spots in gene expression profiling

**DOI:** 10.1038/srep46941

**Published:** 2018-02-12

**Authors:** Carlos P. Roca, Susana I. L. Gomes, Mónica J. B. Amorim, Janeck J. Scott-Fordsmand

Scientific Reports 7: Article number: 42460; 10.1038/srep42460 published online: 03 09 2017; updated: 02 12 2018.

The Acknowledgements section in this Article is incomplete:

“This work was supported by the European Union FP7 projects MODERN (Ref. 309314-2, to C.P.R., J.J.S.-F.), MARINA (Ref. 263215, to J.J.S.-F.), and SUN (Ref. 604305, to C.P.R., S.I.L.G., M.J.B.A., J.J.S.-F.), by FEDER funding through COMPETE (Programa Operacional Factores de Competitividade) and Portugal funding through FCT (Fundação para a Ciência e Tecnologia) within the project bio-CHIP (Refs FCOMP-01-0124-FEDER-041177, FCT EXPL/AAG-MAA/0180/2013, to S.I.L.G., M.J.B.A.), and by a post-doctoral grant (Ref. SFRH/BPD/95775/2013, to S.I.L.G.)”

should read:

“This work was supported by the European Union FP7 projects MODERN (Ref. 309314-2, to C.P.R., J.J.S.-F.), MARINA (Ref. 263215, to J.J.S.-F.), and SUN (Ref. 604305, to C.P.R., S.I.L.G., M.J.B.A., J.J.S.-F.), by FEDER funding through COMPETE (Programa Operacional Factores de Competitividade) and Portugal funding through FCT (Fundação para a Ciência e Tecnologia) within the project bio-CHIP (Refs FCOMP-01-0124-FEDER-041177, FCT EXPL/AAG-MAA/0180/2013, to S.I.L.G., M.J.B.A.), and by a post-doctoral grant (Ref. SFRH/BPD/95775/2013, to S.I.L.G.). Financial support was also received from CESAM (UID/AMB/50017 - POCI-01-0145-FEDER-007638), FCT/MCTES through national funds (PIDDAC), and the co-funding by the FEDER, within the PT2020 Partnership Agreement and Compete 2020.”

