# Supplementary material for: Variation-preserving normalization unveils blind spots in gene expression profiling
Source: Sci Rep. 2017 Mar 9;7:42460. doi: 10.1038/srep42460 (PMC5343588; doi:10.1038/srep42460)
Supplement: Supplementary Information [file srep42460-s1.pdf]

Variation-preserving normalization unveils blind spots in gene expression profiling

Supplementary Information

Carlos P. Roca[1,2,*], Susana I. L. Gomes[3], Mónica J. B. Amorim[3], and Janeck J. Scott-Fordsmand[2,*]

[1]Department of Chemical Engineering, Universitat Rovira i Virgili, 43007 Tarragona, Spain

[2]Department of Bioscience, Aarhus University, 8600 Silkeborg, Denmark

[3]Department of Biology & CESAM, University of Aveiro, 3810-193 Aveiro, Portugal

[*]Correspondence should be addressed to C.P.R. (carlosproca@gmail.com) and J.J.S.-F. (jsf@bios.au.dk)

Contents

1 Supplementary Tables **3**

2 Supplementary Figures **5**

3 Legends of Supplementary Movies **17**

4 Supplementary Mathematical Methods **20**

4.1 Vectorial representation of sample data 20

4.2 Linear decomposition of the normalization problem 22

4.3 Permutation invariance of multivariate data 26

4.4 Standard-vector normalization 32

4.5 Identification of no-variation genes 34

References **36**

1 Supplementary Tables

Supplementary Table S1: Experimental conditions of the toxicity experiment on *E. cryptiticus*, listed in the same order as they appear in each panel of Fig. 1, from left to right.

Condition number	Condition ID	Condition description
1	Ag.AgNO3.EC20.3d	AgNO3 EC20 3 days
2	Ag.AgNO3.EC20.7d	AgNO3 EC20 7 days
3	Ag.AgNO3.EC50.3d	AgNO3 EC50 3 days
4	Ag.AgNO3.EC50.7d	AgNO3 EC50 7 days
5	Ag.Coated.EC20.3d	Ag-NPs PVP-Coated EC20 3 days
6	Ag.Coated.EC20.7d	Ag-NPs PVP-Coated EC20 7 days
7	Ag.Coated.EC50.3d	Ag-NPs PVP-Coated EC50 3 days
8	Ag.Coated.EC50.7d	Ag-NPs PVP-Coated EC50 7 days
9	Ag.NC.EC20.3d	Ag-NPs Non-Coated EC20 3 days
10	Ag.NC.EC20.7d	Ag-NPs Non-Coated EC20 7 days
11	Ag.NC.EC50.3d	Ag-NPs Non-Coated EC50 3 days
12	Ag.NC.EC50.7d	Ag-NPs Non-Coated EC50 7 days
13	Ag.NM300K.EC20.3d	Ag NM300K EC20 3 days
14	Ag.NM300K.EC20.7d	Ag NM300K EC20 7 days
15	Ag.NM300K.EC50.3d	Ag NM300K EC50 3 days
16	Ag.NM300K.EC50.7d	Ag NM300K EC50 7 days
17	Ag.CT.3d	Ag Control 3 days
18	Ag.CT.7d	Ag Control 7 days
19	Ag.CTD.3d	Ag Control Dispersant 3 days
20	Ag.CTD.7d	Ag Control Dispersant 7 days
21	Cu.CuNO3.EC20.3d	CuNO3 EC20 3 days
22	Cu.CuNO3.EC20.7d	CuNO3 EC20 7 days
23	Cu.CuNO3.EC50.3d	CuNO3 EC50 3 days
24	Cu.CuNO3.EC50.7d	CuNO3 EC50 7 days
25	Cu.Cu.NPs.EC20.3d	Cu-NPs EC20 3 days
26	Cu.Cu.NPs.EC20.7d	Cu-NPs EC20 7 days
27	Cu.Cu.NPs.EC50.3d	Cu-NPs EC50 3 days
28	Cu.Cu.NPs.EC50.7d	Cu-NPs EC50 7 days
29	Cu.Cu.Nwires.EC20.3d	Cu-NWires EC20 3 days
30	Cu.Cu.Nwires.EC20.7d	Cu-NWires EC20 7 days
31	Cu.Cu.Nwires.EC50.3d	Cu-NWires EC50 3 days
32	Cu.Cu.Nwires.EC50.7d	Cu-NWires EC50 7 days
33	Cu.Cu.field.EC20.3d	Cu-Field EC20 3 days
34	Cu.Cu.field.EC20.7d	Cu-Field EC20 7 days
35	Cu.Cu.field.EC50.3d	Cu-Field EC50 3 days
36	Cu.Cu.field.EC50.7d	Cu-Field EC50 7 days
37	Cu.CT.3d	Cu Control 3 days
38	Cu.CT.7d	Cu Control 7 days
39	Ni.NiNO3.EC20.3d	NiNO3 EC20 3 days
40	Ni.NiNO3.EC20.7d	NiNO3 EC20 7 days
41	Ni.NiNO3.EC50.3d	NiNO3 EC50 3 days
42	Ni.NiNO3.EC50.7d	NiNO3 EC50 7 days
43	Ni.Ni.NPs.EC20.3d	Ni-NPs EC20 3 days
44	Ni.Ni.NPs.EC20.7d	Ni-NPs EC20 7 days
45	Ni.Ni.NPs.EC50.3d	Ni-NPs EC50 3 days
46	Ni.Ni.NPs.EC50.7d	Ni-NPs EC50 7 days
47	Ni.CT.3d	Ni Control 3 days
48	Ni.CT.7d	Ni Control 7 days
49	Uv.UV.D1.5d	UV Dose 1
50	Uv.UV.D2.5d	UV Dose 2
51	Uv.CT.5d	UV Control

Supplementary Table S2: Treatment vs control comparisons, listed in increasing number of differentially expressed genes (DEGs), obtained for the real *E. crypticus* dataset with SVCD normalization and limma analysis. This is the same order as in Figs. 3a, 4, 5, from left to right.

Comparison number	Treatment ID	Control ID	Treatment description	Number of DEGs
1	Ag.NM300K.EC20.7d	Ag.CTD.7d	Ag NM300K EC20 7 days	2
2	Ni.Ni.NPs.EC20.7d	Ni.CT.7d	Ni-NPs EC20 7 days	7
3	Cu.CuNO3.EC50.7d	Cu.CT.7d	CuNO3 EC50 7 days	26
4	Cu.CuNO3.EC20.3d	Cu.CT.3d	CuNO3 EC20 3 days	27
5	Cu.Cu.NPs.EC20.3d	Cu.CT.3d	Cu-NPs EC20 3 days	31
6	Ag.AgNO3.EC50.7d	Ag.CT.7d	AgNO3 EC50 7 days	33
7	Cu.Cu.NPs.EC20.7d	Cu.CT.7d	Cu-NPs EC20 7 days	33
8	Ag.NM300K.EC20.3d	Ag.CTD.3d	Ag NM300K EC20 3 days	38
9	Ag.NM300K.EC50.3d	Ag.CTD.3d	Ag NM300K EC50 3 days	52
10	Ni.NiNO3.EC20.3d	Ni.CT.3d	NiNO3 EC20 3 days	74
11	Ni.NiNO3.EC20.7d	Ni.CT.7d	NiNO3 EC20 7 days	79
12	Ag.NC.EC20.7d	Ag.CT.7d	Ag-NPs Non-Coated EC20 7 days	106
13	Ni.Ni.NPs.EC50.7d	Ni.CT.7d	Ni-NPs EC50 7 days	107
14	Ag.AgNO3.EC20.7d	Ag.CT.7d	AgNO3 EC20 7 days	113
15	Ag.NC.EC50.7d	Ag.CT.7d	Ag-NPs Non-Coated EC50 7 days	163
16	Ag.NC.EC20.3d	Ag.CT.3d	Ag-NPs Non-Coated EC20 3 days	240
17	Ag.AgNO3.EC50.3d	Ag.CT.3d	AgNO3 EC50 3 days	260
18	Ag.Coated.EC20.7d	Ag.CT.7d	Ag-NPs PVP-Coated EC20 7 days	261
19	Ni.NiNO3.EC50.7d	Ni.CT.7d	NiNO3 EC50 7 days	329
20	Cu.Cu.NPs.EC50.7d	Cu.CT.7d	Cu-NPs EC50 7 days	343
21	Ag.Coated.EC50.7d	Ag.CT.7d	Ag-NPs PVP-Coated EC50 7 days	346
22	Cu.Cu.Nwires.EC50.7d	Cu.CT.7d	Cu-NWires EC50 7 days	383
23	Cu.CuNO3.EC20.7d	Cu.CT.7d	CuNO3 EC20 7 days	393
24	Cu.Cu.Nwires.EC20.7d	Cu.CT.7d	Cu-NWires EC20 7 days	479
25	Cu.CuNO3.EC50.3d	Cu.CT.3d	CuNO3 EC50 3 days	522
26	Ag.AgNO3.EC20.3d	Ag.CT.3d	AgNO3 EC20 3 days	908
27	Ag.Coated.EC20.3d	Ag.CT.3d	Ag-NPs PVP-Coated EC20 3 days	937
28	Ag.NM300K.EC50.7d	Ag.CTD.7d	Ag NM300K EC50 7 days	1,264
29	Ni.Ni.NPs.EC20.3d	Ni.CT.3d	Ni-NPs EC20 3 days	1,464
30	Cu.Cu.field.EC20.7d	Cu.CT.7d	Cu-Field EC20 7 days	1,627
31	Ni.NiNO3.EC50.3d	Ni.CT.3d	NiNO3 EC50 3 days	1,647
32	Uv.UV.D2.5d	Uv.CT.5d	UV Dose 2	1,864
33	Ni.Ni.NPs.EC50.3d	Ni.CT.3d	Ni-NPs EC50 3 days	2,334
34	Cu.Cu.field.EC50.3d	Cu.CT.3d	Cu-Field EC50 3 days	3,570
35	Cu.Cu.field.EC50.7d	Cu.CT.7d	Cu-Field EC50 7 days	4,396
36	Uv.UV.D1.5d	Uv.CT.5d	UV Dose 1	4,745
37	Cu.Cu.NPs.EC50.3d	Cu.CT.3d	Cu-NPs EC50 3 days	5,988
38	Cu.Cu.field.EC20.3d	Cu.CT.3d	Cu-Field EC20 3 days	9,225
39	Ag.Coated.EC50.3d	Ag.CT.3d	Ag-NPs PVP-Coated EC50 3 days	9,478
40	Cu.Cu.Nwires.EC20.3d	Cu.CT.3d	Cu-NWires EC20 3 days	9,751
41	Ag.NC.EC50.3d	Ag.CT.3d	Ag-NPs Non-Coated EC50 3 days	9,884
42	Cu.Cu.Nwires.EC50.3d	Cu.CT.3d	Cu-NWires EC50 3 days	10,285

2 Supplementary Figures

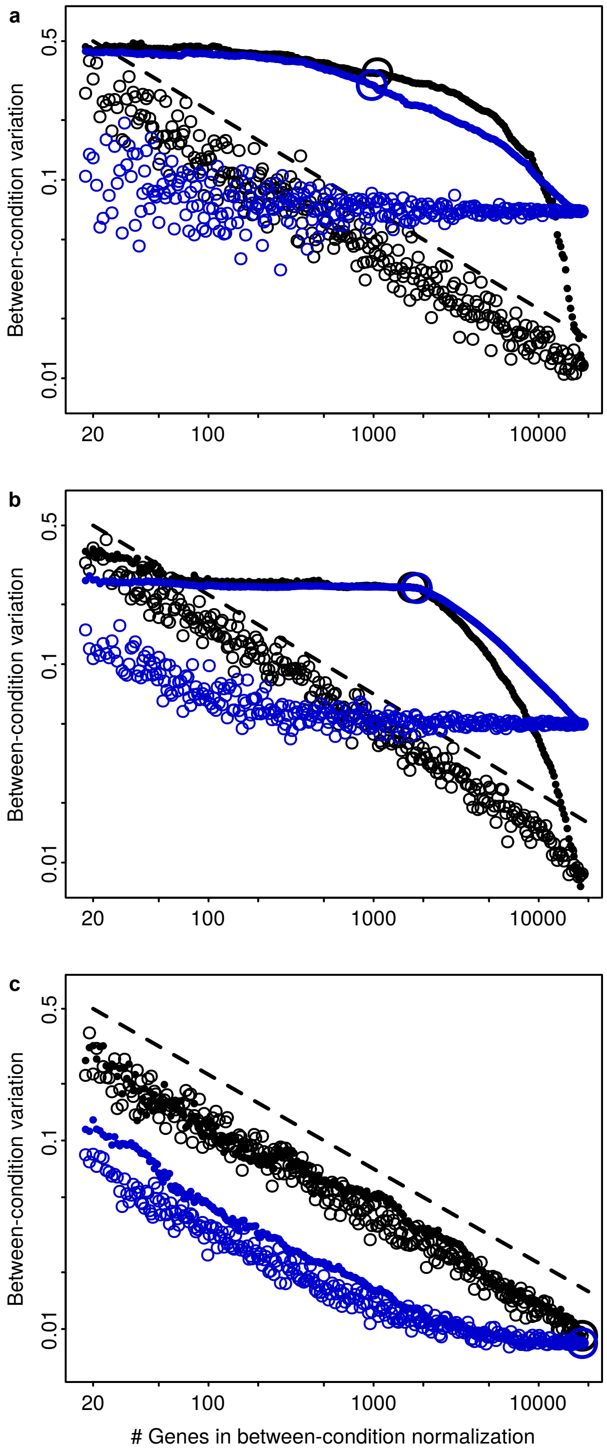

Supplementary Figure S1

Supplementary Figure S1: Representing between-condition variation as the standard deviation of the within-condition median averages (averages of sample medians, for all samples of the condition) produced similar results to those obtained with within-condition mean averages (Fig. 2). Panels show detected variation as a function of the number of genes used in the between-condition normalization, for the real dataset (a), synthetic dataset with differential gene expression (b), and synthetic dataset without differential gene expression (c). Labeling is the same as in Fig. 2. Each point in each panel indicates the variation obtained with one complete normalization (black circles, MedianCD normalization; blue circles, SVCD normalization). Gene were selected in two ways: randomly (empty circles) or in decreasing order of p-values from a test for detecting no-variation genes (filled circles). Big circles show the working points corresponding to the results depicted in Fig. 1j–o. Black dashed lines show references for $n^{-1/2}$ decays, with the same values in all panels.

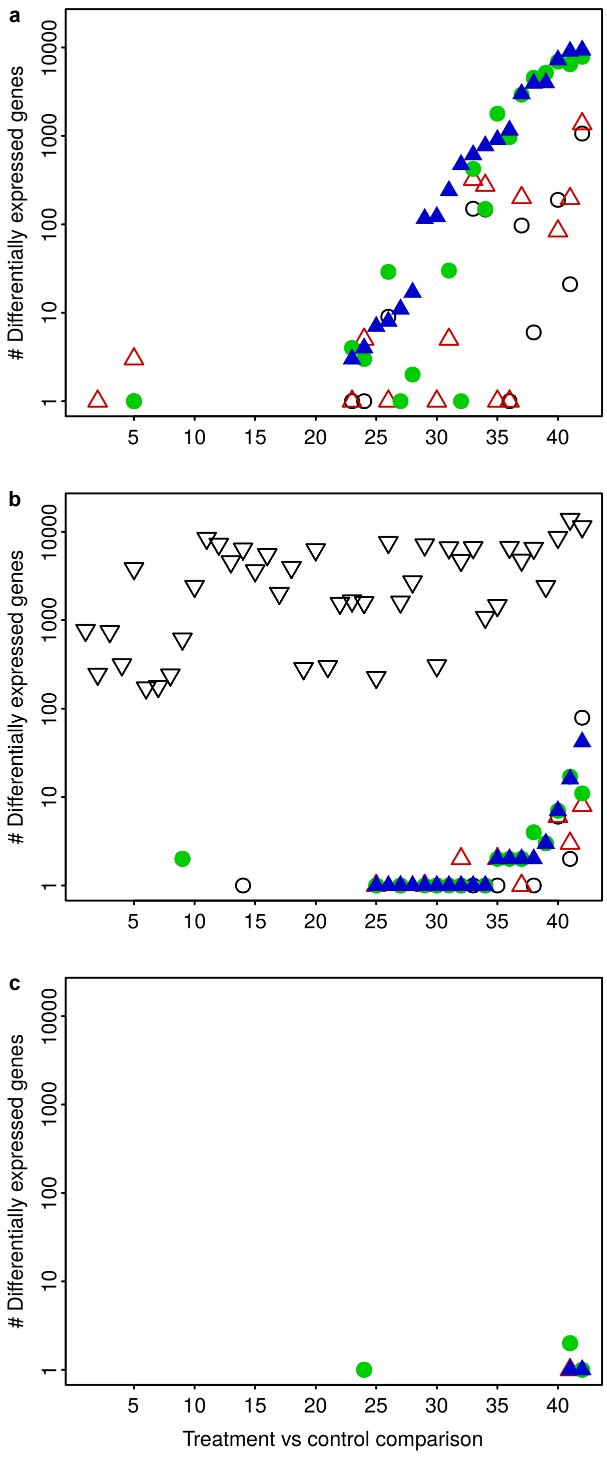

Supplementary Figure S2

Supplementary Figure S2: With t-tests instead of limma analysis (Fig. 3a), MedianCD and SVCD normalization also allowed to detect larger numbers of differentially expressed genes (DEGs), compared to Median and Quantile normalization. Panels show the number of DEGs obtained for the real dataset (a), synthetic dataset with differential gene expression (b), and synthetic dataset without differential gene expression (c). Symbols are the same as in Fig. 3 (empty black circles, Median normalization; empty red up triangles, Quantile normalization; filled green circles, MedianCD normalization; filled blue up triangles, SVCD normalization; empty black down triangles, number of treatment positives (b)). In each panel, treatments are ordered according to the number of DEGs identified with SVCD normalization, increasing from left to right.

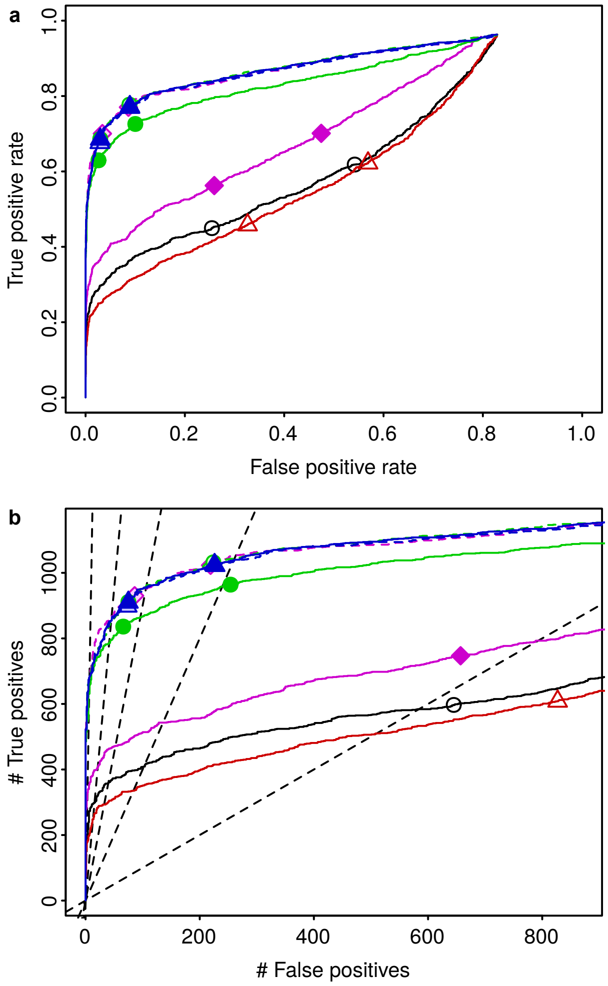

Supplementary Figure S3

Supplementary Figure S3: In the Golden Spike dataset, and without restricting probe sets to those with signal in all samples (Fig. 8), MedianCD and SVCD normalization also allowed the best detection of differential gene expression. Both panels display ROC curves, with the true positive rate versus the false positive rate (a), or the number of true positives versus the number of false positives (b). Each curve shows the results obtained after applying the four normalization methods plus Cyclic Loess normalization (same colors and symbols as in Fig. 8; black curve with empty black circles, Median normalization; red curve with empty red up triangles, Quantile normalization; green curve with filled green circles, MedianCD normalization; blue curve with filled blue up triangles, SVCD normalization; magenta curve with filled magenta diamonds, Cyclic Loess normalization). Dashed curves with lightly filled symbols, overlapping the response of SVCD normalization, show results when the list of known negatives was provided to MedianCD, SVCD, and Cyclic Loess normalization. The two points per normalization method show results when controlling the false discovery rate (FDR) to be below 0.01 (left point) or 0.05 (right point). Dashed lines in (b) show references for actual FDR equal to 0.01, 0.05, 0.1, 0.2, or 0.5 (from left to right). As in Fig. 8, compared to MedianCD and SVCD normalization, the other normalization methods resulted in notably more severe degradation of the FDR.

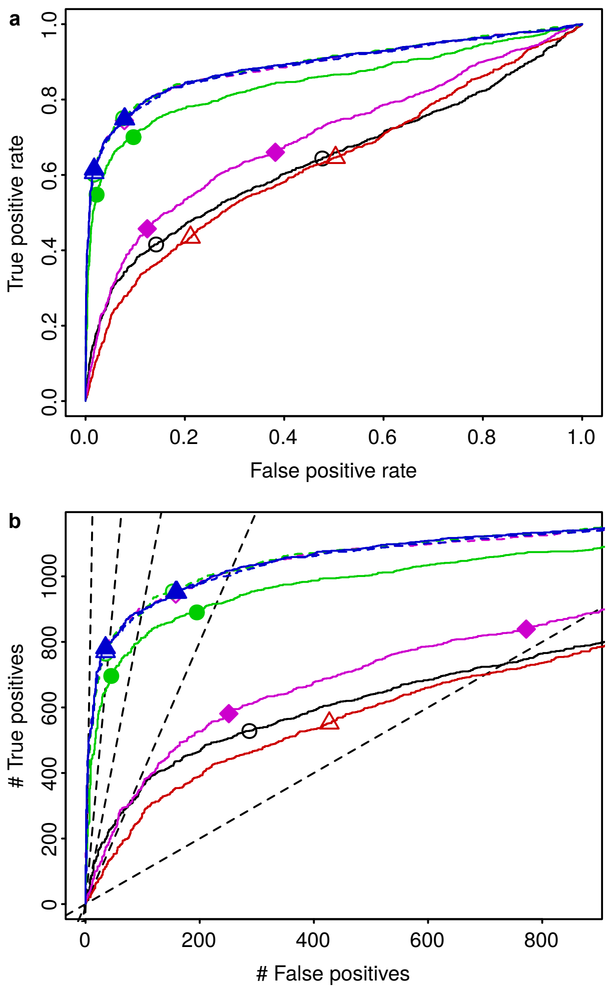

Supplementary Figure S4

Supplementary Figure S4: In the Golden Spike dataset, and with t-tests instead of limma analysis (Fig. 8), MedianCD and SVCD normalization also allowed the best detection of differential gene expression. Both panels display ROC curves, with the true positive rate versus the false positive rate (a), or the number of true positives versus the number of false positives (b). Each curve shows the results obtained after applying the four normalization methods plus Cyclic Loess normalization (same colors and symbols as in Figs. 8, S3; black curve with empty black circles, Median normalization; red curve with empty red up triangles, Quantile normalization; green curve with filled green circles, MedianCD normalization; blue curve with filled blue up triangles, SVCD normalization; magenta curve with filled magenta diamonds, Cyclic Loess normalization). Dashed curves with lightly filled symbols, overlapping the response of SVCD normalization, show results when the list of known negatives was provided to MedianCD, SVCD, and Cyclic Loess normalization. The two points per normalization method show results when controlling the false discovery rate (FDR) to be below 0.01 (left point) or 0.05 (right point). Dashed lines in (b) show references for actual FDR equal to 0.01, 0.05, 0.1, 0.2, or 0.5 (from left to right). Compared to results obtained with limma analysis (Figs. 8, S3), the degradation of FDR was slightly less severe with t-tests. MedianCD and SVCD normalization resulted again in the least degradation of the FDR.

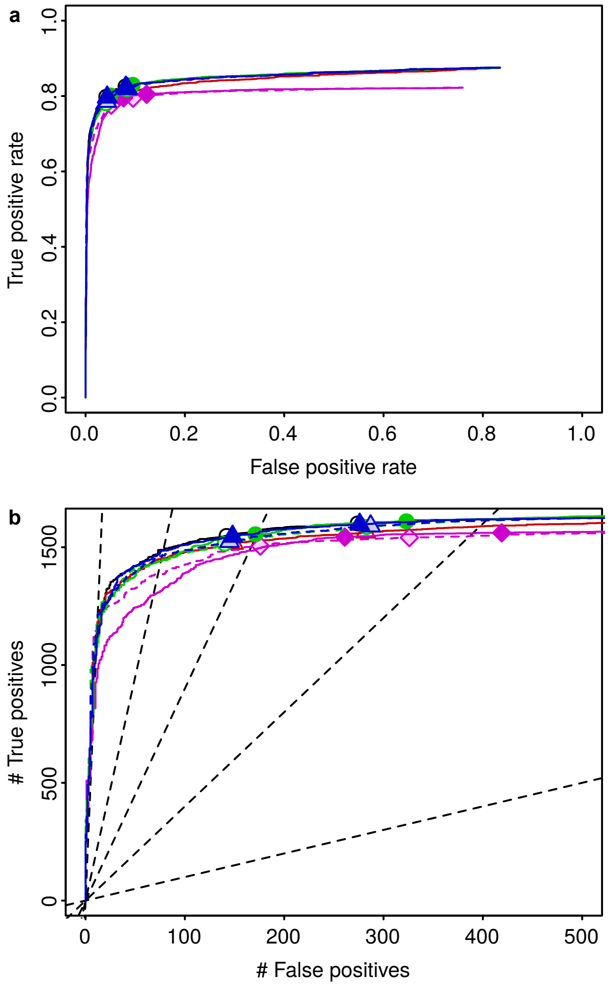

Supplementary Figure S5

Supplementary Figure S5: In the Platinum Spike dataset, and without restricting probe sets to those with signal in all samples (Fig. 9), all normalization methods resulted in similar detection of differential gene expression, with the exception of Cyclic Loess normalization (magenta curve/symbols), whose number of detected positives was slightly smaller. Both panels display ROC curves, with the true positive rate versus the false positive rate (a), or the number of true positives versus the number of false positives (b). Each curve shows the results obtained after applying the four normalization methods plus Cyclic Loess normalization (same colors and symbols as in Fig. 9; black curve with empty black circles, Median normalization; red curve with empty red up triangles, Quantile normalization; green curve with filled green circles, MedianCD normalization; blue curve with filled blue up triangles, SVCD normalization; magenta curve with filled magenta diamonds, Cyclic Loess normalization). Dashed curves with lightly filled symbols show results when the list of known negatives was provided to MedianCD, SVCD, and Cyclic Loess normalization. The two points per normalization method show results when controlling the false discovery rate (FDR) to be below 0.01 (left point) or 0.05 (right point). Dashed lines in (b) show references for actual FDR equal to 0.01, 0.05, 0.1, 0.2, or 0.5 (from left to right). As in Fig. 9, the difference between normalization methods in the resulting degradation of the FDR was smaller for this dataset than for the Golden Spike dataset (Figs. 8, S3, S4).

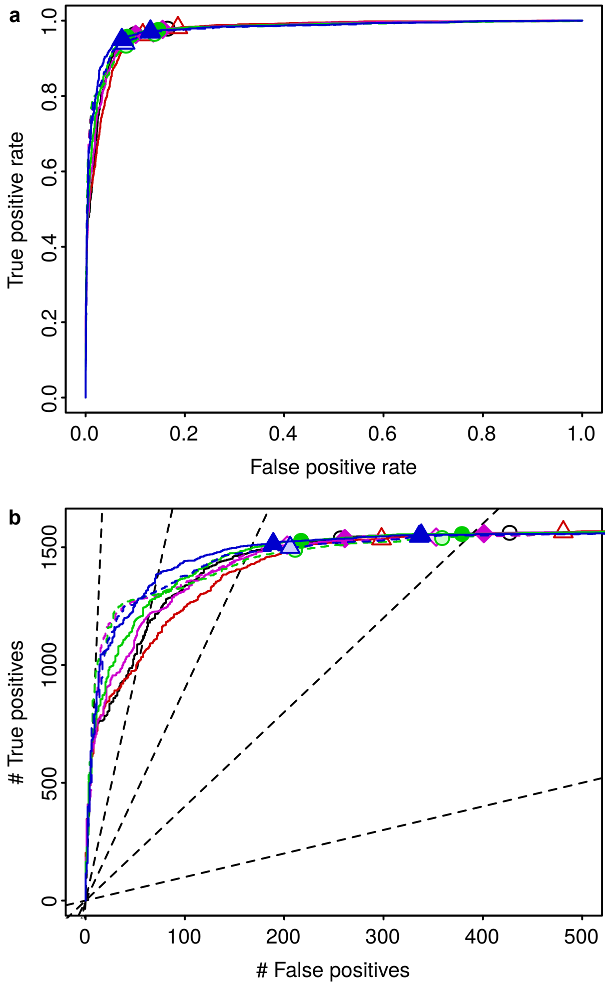

Supplementary Figure S6

Supplementary Figure S6: In the Platinum Spike dataset, and with t-tests instead of limma analysis (Fig. 9), all normalization methods resulted in similar detection of differential gene expression, with MedianCD and SVCD normalization being marginally better. Both panels display ROC curves, with the true positive rate versus the false positive rate (a), or the number of true positives versus the number of false positives (b). Each curve shows the results obtained after applying the four normalization methods plus Cyclic Loess normalization (same colors and symbols as in Figs. 9, S5; black curve with empty black circles, Median normalization; red curve with empty red up triangles, Quantile normalization; green curve with filled green circles, MedianCD normalization; blue curve with filled blue up triangles, SVCD normalization; magenta curve with filled magenta diamonds, Cyclic Loess normalization). Dashed curves with lightly filled symbols show results when the list of known negatives was provided to MedianCD, SVCD, and Cyclic Loess normalization. The two points per normalization method show results when controlling the false discovery rate (FDR) to be below 0.01 (left point) or 0.05 (right point). Dashed lines in (b) show references for actual FDR equal to 0.01, 0.05, 0.1, 0.2, or 0.5 (from left to right). Compared to results obtained with limma analysis (Figs. 9, S5), and in contrast to the Golden Spike dataset (Figs. 8, S3, S4), the degradation of the FDR was slightly more severe with t-tests in this dataset.

3 Legends of Supplementary Movies

Supplementary Movie S1. Example of one within-condition Standard-Vector normalization, for the real (*E. crypticus*) dataset. The movie shows the 14 steps of the convergence of Standard-Vector normalization performed for the condition Ag.NM300K.EC20.3d (exposure to Ag NM300K nanoparticles, with an EC_{50} dose for three days). Left panels show a subset of 10,000 randomly-chosen sample standard vectors, with one gray line per gene, in the plane of residual vectors, i.e. the plane perpendicular to the vector of coordinates $(1, 1, 1)$. The lines labeled s1–s3 indicate the projection of the axes onto this plane, the number 1–3 being the sample number. The red line is the estimated vector of normalization factors at each step, with length $||\text{offset}||$, which results from the bias of the standard vectors towards that direction. Right panels show the polar distribution of vector angles (black solid curve), compared to the distribution of vector angles after all six possible permutations of the sample labels (blue dashed curve). The Watson U^2 statistic provides a measure of the difference between both distributions. In the initial step, there is a large bias towards the first and second sample, compared to the third one. The bias is reduced in each step by subtracting the normalization factor estimate, which makes the distribution of standard vectors more permutationally symmetric and with a correspondingly smaller U^2. After convergence in 14 steps, there is no detectable bias left.

Supplementary Movie S2. Example of one within-condition Standard-Vector normalization, for the synthetic dataset without differential gene expression. The movie displays the Standard-Vector normalization performed for the condition Ag.NM300K.EC20.3d, on the synthetic dataset generated with the standard normal $\mathcal{N}(0, 1)$ as base distribution. Format and labels are the same as in Supplementary Movie S1. Note the uniform distribution of angles after normalizing, which corresponds to a parametric family of probability distributions with spherical symmetry. The corresponding movie for the synthetic dataset with differential gene expression is virtually identical, given that standard vectors are independent of sample averages.

Supplementary Movie S3. Example of one within-condition Standard-Vector normalization, for synthetic log-normal data. The movie shows the standard-vector normalization performed for the condition Ag.NM300K.EC20.3d, on a synthetic dataset generated in the same way as that of Supplementary Movie S2, except for using as base distribution the log-normal $\log \mathcal{N}(0, 0.5^2)$, which has large positive skewness (≈ 1.75). Format and labels are the same as in Supplementary Movies S1, S2. Note that the distribution of standard vector angles after normalizing is not uniform, but it has permutation symmetry.

Supplementary Movie S4. Identification of no-variation genes (non-differentially expressed genes) for the real (*E. crypticus*) dataset. The movie shows the 27 steps of the corresponding between-condition normalization, with SVCD normalization. Both panels show the empirical distribution function of p-values obtained from ANOVA tests on expression levels, per gene and grouped by experimental condition. The left panel shows the complete interval $[0, 1]$, while the right panel depicts the interval close to 1 where the first goodness-of-fit (GoF) test was not rejected. The black portion of the distribution corresponds to p-values at which the GoF test was rejected, the big black circle indicates the first p-value at which the GoF test was not rejected, and the red portion shows the range of p-values whose genes, as a result, were identified as no-variation genes. The dashed blue line and the dotted blue line indicate, respectively, the theoretical distribution function of the uniform distribution and the threshold of the one-sided Kolmogorov-Smirnov test ($\alpha = 0.001$, n equal to the number of p-values for the first GoF test that was not rejected). Convergence criteria was met from steps 18 to 27. These last ten steps ensured stability of the detected set of no-variation genes, by cumulative intersection of the successive sets identified, each one with $\#H_0$ no-variation genes, as shown. The resulting final set had 974 no-variation genes.

Supplementary Movie S5. Identification of no-variation genes for the synthetic dataset with differential gene expression. The movie shows the 15 steps of the corresponding between-condition normalization, with SVCD normalization. Format and labels are the same as in Supplementary Movie S4. Note the similarity with the behavior observed for the real dataset (Supplementary Movie S4).

Supplementary Movie S6. Identification of no-variation genes for the synthetic dataset without differential gene expression. The movie shows the 14 steps of the corresponding between-condition normalization, with SVCD normalization. Format and labels are the same as in Supplementary Movies S4, S5. Note that the distribution of p-values at convergence (steps 5–14) is uniform in the whole interval $[0, 1]$, up to the level detected by the goodness-of-fit test. This corresponds to a dataset with no differentially expressed genes.

4 Supplementary Mathematical Methods

4.1 Vectorial representation of sample data

Let $x_1, \ldots, x_n$ be the samples of n independent and identically distributed random variables $X_1, \ldots, X_n$. Let us represent the samples $x_1, \ldots, x_n$ with the $\mathbb{R}^n$ column vector $\mathbf{x} = (x_1, \ldots, x_n)'$, and let us denote the sample mean by $\bar{x} = \sum_{i=1}^n x_i/n$.

Let us define the $\mathbb{R}^n \to \mathbb{R}^n$ vectorial operators mean $(\overline{\cdot})$ and residual $(\widetilde{\cdot})$, respectively, as

$$\overline{\mathbf{x}} \;=\; (\,\bar{x},\,\ldots\,,\bar{x}\,)' \;=\; \bar{x}\mathbf{1}, \tag{1}$$

$$\widetilde{\mathbf{x}} \;=\; \mathbf{x} - \overline{\mathbf{x}} \;=\; \mathbf{x} - \bar{x}\mathbf{1}, \tag{2}$$

1 being the all-ones column vector of dimension n.

Thus, any sample vector $\mathbf{x} \in \mathbb{R}^n$ can be decomposed as

$$\mathbf{x} \;=\; \overline{\mathbf{x}} + \widetilde{\mathbf{x}}. \tag{3}$$

The mean vector $\overline{\mathbf{x}}$ contains the sample mean, while the residual vector $\widetilde{\mathbf{x}}$ carries the sample variation around the mean.

The vectorial operators mean (1) and residual (2) are linear.

Proposition. For any two sample vectors $\mathbf{x}, \mathbf{y} \in \mathbb{R}^n$ and any two numbers $\alpha, \beta \in \mathbb{R}$,

$$\overline{\alpha\mathbf{x} + \beta\mathbf{y}} \;=\; \alpha\overline{\mathbf{x}} + \beta\overline{\mathbf{y}}, \tag{4}$$

$$\widetilde{\alpha\mathbf{x} + \beta\mathbf{y}} \;=\; \alpha\widetilde{\mathbf{x}} + \beta\widetilde{\mathbf{y}}. \tag{5}$$

Proof. Let us denote $\mathbf{x} = (x_1, \ldots, x_n)'$ and $\mathbf{y} = (y_1, \ldots, y_n)'$.

$$\begin{aligned}
\overline{\alpha\mathbf{x} + \beta\mathbf{y}} &= \frac{\sum_{i=1}^n (\alpha x_i + \beta y_i)}{n}\mathbf{1} \;=\; \alpha\frac{\sum_{i=1}^n x_i}{n}\mathbf{1} + \beta\frac{\sum_{i=1}^n y_i}{n}\mathbf{1} \;=\; \alpha\overline{\mathbf{x}} + \beta\overline{\mathbf{y}}, \\
\widetilde{\alpha\mathbf{x} + \beta\mathbf{y}} &= \alpha\mathbf{x} + \beta\mathbf{y} - \overline{\alpha\mathbf{x} + \beta\mathbf{y}} \;=\; \alpha\mathbf{x} + \beta\mathbf{y} - (\alpha\overline{\mathbf{x}} + \beta\overline{\mathbf{y}}), \\
&= \alpha(\mathbf{x} - \overline{\mathbf{x}}) + \beta(\mathbf{y} - \overline{\mathbf{y}}) \;=\; \alpha\widetilde{\mathbf{x}} + \beta\widetilde{\mathbf{y}}. \quad \square
\end{aligned}$$

An essential property of the mean and residual vectors is that they belong to subspaces that are orthogonal complements[1]. Hence, for any sample vector $\mathbf{x} \in \mathbb{R}^n$, the mean vector $\overline{\mathbf{x}}$ belongs to the subspace of dimension 1 spanned by the unit vector $\widehat{\mathbf{1}} = \mathbf{1}/\sqrt{n}$, while the residual vector $\widetilde{\mathbf{x}}$ belongs to the $(n-1)$-dimensional hyperplane orthogonal to $\widehat{\mathbf{1}}$.

The lengths of the mean vector and residual vector are equal, up to a scaling factor, to the sample mean and sample standard deviation, respectively. For a set of samples $x_1, \ldots, x_n$, where $n \geq 2$, let us denote the sample mean as before by $\bar{x} = \sum_{i=1}^n x_i/n$, and the sample variance as $s_x^2 = \sum_{i=1}^n (x_i - \bar{x})^2/(n-1)$. Then, the lengths of the mean and residual vectors obtained from the sample vector $\mathbf{x} = (x_1, \ldots, x_n)'$ are

$$\|\overline{\mathbf{x}}\| = \sqrt{n\,\bar{x}^2} = \sqrt{n}\,|\bar{x}|, \tag{6}$$

$$\|\widetilde{\mathbf{x}}\| = \sqrt{\sum_{i=1}^n (x_i - \bar{x})^2} = \sqrt{n-1}\,s_x. \tag{7}$$

Finally, let us define the standard vector of the sample vector $\mathbf{x} = (x_1, \ldots, x_n)'$ $(n \geq 2)$, as

$$\text{stdvec}(\mathbf{x}) = \sqrt{n-1}\frac{\widetilde{\mathbf{x}}}{\|\widetilde{\mathbf{x}}\|}, \tag{8}$$

whenever $\widetilde{\mathbf{x}} \neq \mathbf{0}$, or otherwise as $\text{stdvec}(\mathbf{x}) = \mathbf{0}$. $\mathbf{0}$ is the all-zeros column vector of dimension n.

For a given number of samples n, all the non-zero standard vectors belong to the $(n-2)$-sphere of radius $\sqrt{n-1}$, embedded in the $(n-1)$-dimensional hyperplane perpendicular to $\widehat{\mathbf{1}}$. Besides, all the components of a standard vector are equal to the corresponding standardized samples,

$$\sqrt{n-1}\frac{\widetilde{x}_i}{\|\widetilde{\mathbf{x}}\|} = \frac{x_i - \bar{x}}{s_x}. \tag{9}$$

For the degenerate case of having only two samples $(n = 2)$, the only possible values of a non-zero standard vector are $\pm(\,1/\sqrt{2},\, -1/\sqrt{2}\,)'$.

4.2 Linear decomposition of the normalization problem

Let us consider a gene expression dataset, with g genes and c experimental conditions. Each condition k has s_k samples. The total number of samples is $s = \sum_{k=1}^{c} s_k$.

Let us denote the *observed* expression level of gene j in the sample i of condition k by $y_{ij}^{(k)}$. We assume that the observed level $y_{ij}^{(k)}$ is equal, in the usual $\log_2$-scale, to the addition of the normalization factor $a_i^{(k)}$ to the *true* gene expression level $x_{ij}^{(k)}$,

$$y_{ij}^{(k)} \;=\; x_{ij}^{(k)} + a_i^{(k)}. \tag{10}$$

Solving the *normalization problem* amounts to finding the normalization factors $a_i^{(k)}$ from the observed values $y_{ij}^{(k)}$. The normalization factors can be understood as sample-wide changes in the concentration of mRNA molecules by multiplicative factors equal to $2^{a_i^{(k)}}$. These changes are caused by technical reasons in the assay and are independent of the biological variation in the true levels $x_{ij}^{(k)}$.

Let us represent the true and observed expression levels, $x_{ij}^{(k)}$ and $y_{ij}^{(k)}$, of gene j in the samples $i = 1 \ldots s_k$ of condition k, by the s_k-dimensional vectors

$$\mathbf{x}_j^{(k)} \;=\; (\, x_{1j}^{(k)}, \ldots, x_{s_k j}^{(k)}\,)', \tag{11}$$

$$\mathbf{y}_j^{(k)} \;=\; (\, y_{1j}^{(k)}, \ldots, y_{s_k j}^{(k)}\,)'. \tag{12}$$

Let us also represent the unknown normalization factors of condition k by the s_k-dimensional vector

$$\mathbf{a}^{(k)} \;=\; (\, a_1^{(k)}, \ldots, a_{s_k}^{(k)}\,)'. \tag{13}$$

From (10)–(13), the normalization problem can be written in vectorial form as

$$\mathbf{y}_j^{(k)} \;=\; \mathbf{x}_j^{(k)} + \mathbf{a}^{(k)}. \tag{14}$$

Applying the vectorial operators mean (1) and residual (2), we obtain

$$\overline{\mathbf{y}}_j^{(k)} \;=\; \overline{\mathbf{x}}_j^{(k)} + \overline{\mathbf{a}}^{(k)}, \tag{15}$$

$$\widetilde{\mathbf{y}}_j^{(k)} \;=\; \widetilde{\mathbf{x}}_j^{(k)} + \widetilde{\mathbf{a}}^{(k)}. \tag{16}$$

The residual-vector equations (16) correspond to the c within-condition normalizations. Each within-condition normalization uses the equations (16) particular to a condition k, for the subset of genes $\mathcal{G}_k \subseteq \{1, \ldots, g\}$ that have expression level available and of enough quality in that experimental condition.

Let us denote the condition means for each gene as

$$\overline{x}_j^{(k)} = \frac{\sum_{i=1}^{s_k} x_{ij}^{(k)}}{s_k}, \tag{17}$$

$$\overline{y}_j^{(k)} = \frac{\sum_{i=1}^{s_k} y_{ij}^{(k)}}{s_k}, \tag{18}$$

$$\overline{a}^{(k)} = \frac{\sum_{i=1}^{s_k} a_i^{(k)}}{s_k}, \tag{19}$$

so that

$$\overline{\mathbf{x}}_j^{(k)} = \overline{x}_j^{(k)} \mathbf{1}_{s_k}, \tag{20}$$

$$\overline{\mathbf{y}}_j^{(k)} = \overline{y}_j^{(k)} \mathbf{1}_{s_k}, \tag{21}$$

$$\overline{\mathbf{a}}^{(k)} = \overline{a}^{(k)} \mathbf{1}_{s_k}, \tag{22}$$

$\mathbf{1}_{s_k}$ being the all-ones column vector of dimension s_k.

Then, the mean-vector equations (15) can be written as

$$\overline{y}_j^{(k)} \mathbf{1}_{s_k} = \overline{x}_j^{(k)} \mathbf{1}_{s_k} + \overline{a}^{(k)} \mathbf{1}_{s_k}, \tag{23}$$

so they reduce to the scalar equations

$$\overline{y}_j^{(k)} = \overline{x}_j^{(k)} + \overline{a}^{(k)}. \tag{24}$$

Let us define the vectors of conditions means as

$$\mathbf{x}_j^* = (\overline{x}_j^{(1)}, \ldots, \overline{x}_j^{(c)})', \tag{25}$$

$$\mathbf{y}_j^* = (\overline{y}_j^{(1)}, \ldots, \overline{y}_j^{(c)})', \tag{26}$$

$$\mathbf{a}^* = (\overline{a}^{(1)}, \ldots, \overline{a}^{(c)})', \tag{27}$$

and let us express the condition-mean equations in vectorial form as

$$\mathbf{y}_j^* = \mathbf{x}_j^* + \mathbf{a}^*. \tag{28}$$

Applying again the mean and variance operators, we obtain

$$\overline{\mathbf{y}}_j^* = \overline{\mathbf{x}}_j^* + \overline{\mathbf{a}}^*, \tag{29}$$

$$\widetilde{\mathbf{y}}_j^* = \widetilde{\mathbf{x}}_j^* + \widetilde{\mathbf{a}}^*. \tag{30}$$

The residual-vector equations on condition means (30) correspond to the single between-condition normalization, in a similar way as (16) do for the each of the within-condition normalizations. There is one equation (30) per gene. The only equations used in the between-condition normalization are those of the subset of genes $\mathcal{G}^* \subseteq \{1, \ldots, g\}$ that show no evidence of variation across experimental conditions, according to a statistical test.

Given that $\overline{\mathbf{a}}^* = \overline{a}^* \mathbf{1}_c$, (29) has the only unknown $\overline{a}^*$. The meaning of $\overline{a}^*$ is a conversion factor between the scale the true and observed expression levels. This factor depends on the technology used to measure the expression levels and finding it is out of the scope of the normalization problem. Therefore, without loss of generality, we assume $\overline{a}^* = 0$, so

$$\overline{\mathbf{a}}^* = \mathbf{0}_c, \tag{31}$$

$$\mathbf{a}^* = \widetilde{\mathbf{a}}^*. \tag{32}$$

The solution of the between-condition normalization, $\widetilde{\mathbf{a}}^*$, allows to find the mean vectors of the normalization factors $\overline{\mathbf{a}}^{(k)}$, via (22), (27) and (32). The within-condition normalizations yield the residual vectors $\widetilde{\mathbf{a}}^{(k)}$. The complete solution to the normalization problem is finally obtained, for each condition k, with

$$\mathbf{a}^{(k)} = \overline{\mathbf{a}}^{(k)} + \widetilde{\mathbf{a}}^{(k)}. \tag{33}$$

Thus, the original normalization problem (14) has been divided in $c+1$ normalization subproblems on residual vectors, stated by (16) and (30). In fact, this linear decomposition is possible for any partition of the set of s samples. The choice of the partition as the one defined by the experimental conditions is motivated by the need to control the biological variation among the genes used in each normalization. All the $c+1$ normalizations face the same kind of *normalization of residuals problem*, which we define in general as follows.

Normalization of Residuals Problem. Let y_{ij} be the i-th observed value of feature j, in a dataset with $n \geq 2$ observations for each of the m features. The observed values y_{ij} are equal to the true values x_{ij} plus the normalization factors a_i, which are constant across features. In vectorial form, there are m equations

$$\mathbf{y}_j \;=\; \mathbf{x}_j + \mathbf{a}, \tag{34}$$

where the vectors belong to $\mathbb{R}^n$. As a consequence

$$\widetilde{\mathbf{y}}_j \;=\; \widetilde{\mathbf{x}}_j + \widetilde{\mathbf{a}}. \tag{35}$$

Solving the *normalization of residuals problem* amounts to finding the residual vector of normalization factors $\widetilde{\mathbf{a}}$ from the observed residual vectors $\widetilde{\mathbf{y}}_j$. In the within-condition normalizations, the features are gene expression levels, with one observation per sample of the corresponding experimental condition. In the between-condition normalization, the features are means of gene expression levels, with one observation per condition.

There is, however, an additional requirement imposed by the methods with which we propose to solve the between-condition normalization. We would like to consider the condition means $\overline{x}_j^{(k)}$ in (24) as sample data across conditions. This only holds when all the conditions have the same number of samples. Otherwise, we balance the condition means so that they result from the same number of samples in all conditions, according to the procedure described in the following.

Let s^* be the minimum number of samples across conditions, $s^* = \min\{s_1, \ldots, s_c\}$. Let $\mathcal{S}_j^{(k)}$ be independent random samples (without replacement) of size s^* from the set of indexes $\{1, \ldots, s_k\}$, with one sample per gene j and condition k. Then, the balanced

condition means are defined as

$$\overline{x}_j^{(k)*} = \frac{\sum_{i \in \mathcal{S}_j^{(k)}} x_{ij}^{(k)}}{s^*}, \tag{36}$$

$$\overline{y}_j^{(k)*} = \frac{\sum_{i \in \mathcal{S}_j^{(k)}} y_{ij}^{(k)}}{s^*}, \tag{37}$$

$$\overline{a}_j^{(k)*} = \frac{\sum_{i \in \mathcal{S}_j^{(k)}} a_i^{(k)}}{s^*}. \tag{38}$$

From (10), the balanced condition means verify a relationship similar to (24),

$$\overline{y}_j^{(k)*} = \overline{x}_j^{(k)*} + \overline{a}_j^{(k)*}. \tag{39}$$

Moreover, the average of $\overline{a}_j^{(k)*}$ across the sampling subsets $\mathcal{S}_j^{(k)}$ is equal to the unknown $\overline{a}^{(k)}$. This implies that (39) are, on average, equivalent to (24). Hence, we use the following vectors of balanced conditions means

$$\mathbf{x}_j^* = (\, \overline{x}_j^{(1)*}, \ldots, \overline{x}_j^{(c)*}\,), \tag{40}$$

$$\mathbf{y}_j^* = (\, \overline{y}_j^{(1)*}, \ldots, \overline{y}_j^{(c)*}\,), \tag{41}$$

instead of (25), (26), in order to build the condition-mean equations (28). This balancing of the condition means is only required when the experimental conditions have different number of samples.

4.3 Permutation invariance of multivariate data

Let x_{ij} and y_{ij} be, respectively, the true and observed values of a dataset with n observations of m features, as defined in the *normalization of residuals problem* above.

We have assumed that the n true values $x_{1j}, \ldots, x_{nj}$ of feature j are samples of independent and identically distributed random variables $X_{1j}, \ldots, X_{nj}$. These random variables can be represented with the random vector $\mathbf{X}_j = (X_{1j}, \ldots, X_{nj})'$, carried by the probability

space $(\Omega, \mathcal{F}, \mathrm{P})$ and with induced space $(\mathbb{R}^n, \mathbb{B}^n, \mathbb{P})$. Let us define the random vectors $\overline{\mathbf{X}}_j$ and $\widetilde{\mathbf{X}}_j$ with the vectorial operators mean (1) and residual (2), respectively,

$$\overline{X}_j = \sum_{i=1}^{n} \frac{X_{ij}}{n}, \tag{42}$$

$$\overline{\mathbf{X}}_j = (\,\overline{X}_j, \ldots, \overline{X}_j\,)' = \overline{X}_j \mathbf{1}, \tag{43}$$

$$\widetilde{\mathbf{X}}_j = \mathbf{X}_j - \overline{\mathbf{X}}_j = \mathbf{X}_j - \overline{X}_j \mathbf{1}. \tag{44}$$

$\mathbf{X}_j = \overline{\mathbf{X}}_j + \widetilde{\mathbf{X}}_j$ holds for any random vector $\mathbf{X}_j$, as well as the other properties presented above. Let us assume that $\mathrm{E}(\,\|\mathbf{X}_j\|\,) < \infty$ and that $\mathrm{P}(\,\|\widetilde{\mathbf{X}}_j\| = 0\,) = 0$, which imply that $\widetilde{\mathbf{X}}_j/\|\widetilde{\mathbf{X}}_j\|$ has length 1 almost surely.

The standard random vector $\sqrt{n-1}\,\widetilde{\mathbf{X}}_j/\|\widetilde{\mathbf{X}}_j\|$ is a pivotal quantity, where the location (mean) and scale (standard deviation) of feature j have been removed. The probability distribution of $\widetilde{\mathbf{X}}_j/\|\widetilde{\mathbf{X}}_j\|$ across the remaining degrees of freedom over the unit $(n-2)$-sphere is governed by the parametric family of the random variables $X_{1j}, \ldots, X_{nj}$. Moreover, the independence and identity of distribution across the n observations implies that the distribution of $\mathbf{X}_j$ is *exchangeable*, i.e. invariant with respect to permutations of the observation labels. As a result, $\widetilde{\mathbf{X}}_j/\|\widetilde{\mathbf{X}}_j\|$ is also permutation invariant, which geometrically corresponds to symmetries with respect to the $n!$ permutations of the axes in the n-dimensional space of random vectors, projected onto the $(n-1)$-dimensional hyperplane of residual vectors.

Residual vectors and standard vectors have been widely studied, especially in relation to elliptically symmetric distributions and linear models[2,3], and to the invariances of probability distributions[4]. Here, we consider these vectors from the viewpoint of the problem of normalizing multivariate data, and its relationship with permutation invariance.

It is well know that, for a multivariate distribution with independent and identically distributed components, the expected value of the standard vector is zero[1], given that it is so for each component. We prove this here for completeness, and to show that it is also a necessary consequence of the permutation invariance of the distribution.

Proposition. The expected value of any true (i.e. without normalization issues) standard vector is zero. If the $n \geq 2$ samples of feature j are independent and identically distributed, then

$$\mathrm{E}\left(\sqrt{n-1}\,\frac{\widetilde{\mathbf{X}}_j}{\|\widetilde{\mathbf{X}}_j\|}\right) \;=\; \mathbf{0}. \tag{45}$$

Proof. Let $\mathcal{P}_n$ be the set of all the permutation matrices in $\mathbb{R}^{n\times n}$. Then, for any $P \in \mathcal{P}_n$, $\widetilde{\mathbf{X}}_j/\|\widetilde{\mathbf{X}}_j\|$ is equal in distribution to $P\,\widetilde{\mathbf{X}}_j/\|\widetilde{\mathbf{X}}_j\|$. This implies that

$$\mathrm{E}\left(\frac{\widetilde{\mathbf{X}}_j}{\|\widetilde{\mathbf{X}}_j\|}\right) \;=\; \mathrm{E}\left(P\frac{\widetilde{\mathbf{X}}_j}{\|\widetilde{\mathbf{X}}_j\|}\right) \;=\; P\,\mathrm{E}\left(\frac{\widetilde{\mathbf{X}}_j}{\|\widetilde{\mathbf{X}}_j\|}\right).$$

The only vectors that are invariant with respect to all possible permutations are those that have all components identical. Therefore, $\mathrm{E}(\,\widetilde{\mathbf{X}}_j/\|\widetilde{\mathbf{X}}_j\|\,) = \alpha\widehat{\mathbf{1}}$, with $\alpha \in \mathbb{R}$. However, $\widetilde{\mathbf{X}}_j'\widehat{\mathbf{1}} = 0$, so that $\alpha = \mathrm{E}(\,\widetilde{\mathbf{X}}_j/\|\widetilde{\mathbf{X}}_j\|\,)'\,\widehat{\mathbf{1}} = 0$. Hence $\mathrm{E}(\,\widetilde{\mathbf{X}}_j/\|\widetilde{\mathbf{X}}_j\|\,) = \mathbf{0}$. □

For each true random vector $\mathbf{X}_j$, there is an observed random vector $\mathbf{Y}_j = \mathbf{X}_j + \mathbf{A}$, where $\mathbf{A}$ is the random vector of normalization factors. The random vectors $\mathbf{X}_j$ and $\mathbf{A}$ are independent, representing biological and technical variation, respectively. Therefore, and without loss of generality, we assume in what follows a fixed vector of normalization factors $\mathbf{a}$, i.e. we condition on the event $\{\mathbf{A} = \mathbf{a}\,\}$. We also assume that $\mathrm{P}(\,\|\widetilde{\mathbf{Y}}_j\| = 0\,) = 0$, which implies that $\widetilde{\mathbf{Y}}_j/\|\widetilde{\mathbf{Y}}_j\|$ has length 1 almost surely.

In contrast to the true standard vector $\sqrt{n-1}\,\widetilde{\mathbf{X}}_j/\|\widetilde{\mathbf{X}}_j\|$, the observed standard vector $\sqrt{n-1}\,\widetilde{\mathbf{Y}}_j/\|\widetilde{\mathbf{Y}}_j\|$ is biased toward the direction of $\widetilde{\mathbf{a}}$, with the result that the expected value is not zero.

Proposition. If the $n \geq 2$ samples of feature j are independent and identically distributed, whenever $\widetilde{\mathbf{a}} \neq \mathbf{0}$,

$$\mathrm{E}\left(\sqrt{n-1}\,\frac{\widetilde{\mathbf{Y}}_j}{\|\widetilde{\mathbf{Y}}_j\|}\right) \;\neq\; \mathbf{0}. \tag{46}$$

When $n = 2$, there is the additional requirement that $\mathrm{P}(\,\|\widetilde{\mathbf{X}}_i\| < \|\widetilde{\mathbf{a}}\|\,) > 0$. This threshold of detection only occurs for the degenerate case of $n = 2$.

Proof. Let us consider the projection of $\widetilde{\mathbf{Y}}_j/\|\widetilde{\mathbf{Y}}_j\|$ on $\widetilde{\mathbf{a}}$, compared to the projection of $\widetilde{\mathbf{X}}_j/\|\widetilde{\mathbf{X}}_j\|$.

When the vectors $\widetilde{\mathbf{X}}_j$ and $\widetilde{\mathbf{a}}$ are collinear,

$$\frac{\widetilde{\mathbf{X}}_j'\,\widetilde{\mathbf{a}}}{\|\widetilde{\mathbf{X}}_j\|\,\|\widetilde{\mathbf{a}}\|} = \pm 1, \quad \text{and} \quad \frac{\widetilde{\mathbf{Y}}_j'\,\widetilde{\mathbf{a}}}{\|\widetilde{\mathbf{Y}}_j\|\,\|\widetilde{\mathbf{a}}\|} = \pm 1,$$

with

$$\frac{\widetilde{\mathbf{Y}}_j'\,\widetilde{\mathbf{a}}}{\|\widetilde{\mathbf{Y}}_j\|\,\|\widetilde{\mathbf{a}}\|} \geq \frac{\widetilde{\mathbf{X}}_j'\,\widetilde{\mathbf{a}}}{\|\widetilde{\mathbf{X}}_j\|\,\|\widetilde{\mathbf{a}}\|}.$$

This is the only case when $n = 2$. The additional requirement ensures that, for $n = 2$,

$$\mathrm{P}\left(\frac{\widetilde{\mathbf{Y}}_j'\,\widetilde{\mathbf{a}}}{\|\widetilde{\mathbf{Y}}_j\|\,\|\widetilde{\mathbf{a}}\|} > \frac{\widetilde{\mathbf{X}}_j'\,\widetilde{\mathbf{a}}}{\|\widetilde{\mathbf{X}}_j\|\,\|\widetilde{\mathbf{a}}\|}\right) > 0,$$

which implies

$$\mathrm{E}\left(\frac{\widetilde{\mathbf{Y}}_j'\,\widetilde{\mathbf{a}}}{\|\widetilde{\mathbf{Y}}_j\|\,\|\widetilde{\mathbf{a}}\|}\right) > \mathrm{E}\left(\frac{\widetilde{\mathbf{X}}_j'\,\widetilde{\mathbf{a}}}{\|\widetilde{\mathbf{X}}_j\|\,\|\widetilde{\mathbf{a}}\|}\right).$$

Otherwise, when $n > 2$ and the vectors $\widetilde{\mathbf{X}}_j$ and $\widetilde{\mathbf{a}}$ are not collinear, they lie on a plane. The vector $\widetilde{\mathbf{Y}}_j = \widetilde{\mathbf{X}}_j + \widetilde{\mathbf{a}}$ is the diagonal of the parallelogram defined by $\widetilde{\mathbf{X}}_j$ and $\widetilde{\mathbf{a}}$. Hence the angle between $\widetilde{\mathbf{Y}}_j$ and $\widetilde{\mathbf{a}}$ is strictly less than the angle between $\widetilde{\mathbf{X}}_j$ and $\widetilde{\mathbf{a}}$, so the cosine of the angle is strictly greater. Thus,

$$\frac{\widetilde{\mathbf{Y}}_j'\,\widetilde{\mathbf{a}}}{\|\widetilde{\mathbf{Y}}_j\|\,\|\widetilde{\mathbf{a}}\|} > \frac{\widetilde{\mathbf{X}}_j'\,\widetilde{\mathbf{a}}}{\|\widetilde{\mathbf{X}}_j\|\,\|\widetilde{\mathbf{a}}\|}.$$

Due to the permutation symmetries in the distribution of $\widetilde{\mathbf{X}}_j/\|\widetilde{\mathbf{X}}_j\|$, when $n > 2$ the vector $\widetilde{\mathbf{X}}_j$ has non-zero probability of being not collinear with $\widetilde{\mathbf{a}}$, i.e. $\mathrm{P}(\,|\widetilde{\mathbf{X}}_j'\,\widetilde{\mathbf{a}}| < 1\,) > 0$.

Therefore,

$$\mathrm{P}\left(\frac{\widetilde{\mathbf{Y}}_j'\,\widetilde{\mathbf{a}}}{\|\widetilde{\mathbf{Y}}_j\|\,\|\widetilde{\mathbf{a}}\|} > \frac{\widetilde{\mathbf{X}}_j'\,\widetilde{\mathbf{a}}}{\|\widetilde{\mathbf{X}}_j\|\,\|\widetilde{\mathbf{a}}\|}\right) > 0,$$

which again implies

$$\mathrm{E}\left(\frac{\widetilde{\mathbf{Y}}_j'\,\widetilde{\mathbf{a}}}{\|\widetilde{\mathbf{Y}}_j\|\,\|\widetilde{\mathbf{a}}\|}\right) > \mathrm{E}\left(\frac{\widetilde{\mathbf{X}}_j'\,\widetilde{\mathbf{a}}}{\|\widetilde{\mathbf{X}}_j\|\,\|\widetilde{\mathbf{a}}\|}\right).$$

Finally,

$$\left\|\mathrm{E}\left(\frac{\widetilde{\mathbf{Y}}_j}{\|\widetilde{\mathbf{Y}}_j\|}\right)\right\| \geq \mathrm{E}\left(\frac{\widetilde{\mathbf{Y}}_j}{\|\widetilde{\mathbf{Y}}_j\|}\right)'\frac{\widetilde{\mathbf{a}}}{\|\widetilde{\mathbf{a}}\|} > \mathrm{E}\left(\frac{\widetilde{\mathbf{X}}_j}{\|\widetilde{\mathbf{X}}_j\|}\right)'\frac{\widetilde{\mathbf{a}}}{\|\widetilde{\mathbf{a}}\|} = 0. \quad \square$$

As a consequence, the *normalization of residuals problem* may be restated as the problem of finding the normalization factors $\widetilde{\mathbf{a}}$ from the observed vectors $\widetilde{\mathbf{y}}_j$, such that the standard vectors $\sqrt{n-1}\,(\widetilde{\mathbf{y}}_j - \widetilde{\mathbf{a}})/\|\widetilde{\mathbf{y}}_j - \widetilde{\mathbf{a}}\|$ are invariant against permutations of the observation labels. Or equivalently, such that the standard vectors $\sqrt{n-1}\,(\widetilde{\mathbf{y}}_j - \widetilde{\mathbf{a}})/\|\widetilde{\mathbf{y}}_j - \widetilde{\mathbf{a}}\|$ have zero mean. The following property provides an approach to the solution.

Proposition. Whenever $\widetilde{\mathbf{a}} \neq \mathbf{0}$, the component of the expected value of $\widetilde{\mathbf{Y}}_j/\|\widetilde{\mathbf{Y}}_j\|$ parallel to $\widetilde{\mathbf{a}}$ verifies

$$0 \;<\; \mathrm{E}\left(\frac{\widetilde{\mathbf{Y}}_j}{\|\widetilde{\mathbf{Y}}_j\|}\right)'\frac{\widetilde{\mathbf{a}}}{\|\widetilde{\mathbf{a}}\|} \;<\; \mathrm{E}\left(\frac{1}{\|\widetilde{\mathbf{Y}}_j\|}\right)\|\widetilde{\mathbf{a}}\|. \tag{47}$$

As in (46), when $n=2$ we also assume that $\mathrm{P}(\,\|\widetilde{\mathbf{X}}_j\| < \|\widetilde{\mathbf{a}}\|\,) > 0$.

Proof. The first inequality holds from the previous proof. Concerning the second inequality, let us consider

$$\frac{\widetilde{\mathbf{Y}}_j'\,\widetilde{\mathbf{a}}}{\|\widetilde{\mathbf{Y}}_j\|\,\|\widetilde{\mathbf{a}}\|} = \frac{(\widetilde{\mathbf{X}}_j + \widetilde{\mathbf{a}})'\,\widetilde{\mathbf{a}}}{\|\widetilde{\mathbf{X}}_j + \widetilde{\mathbf{a}}\|\,\|\widetilde{\mathbf{a}}\|} = \frac{\|\widetilde{\mathbf{X}}_j\|}{\|\widetilde{\mathbf{X}}_j + \widetilde{\mathbf{a}}\|}\,\frac{\widetilde{\mathbf{X}}_j'\,\widetilde{\mathbf{a}}}{\|\widetilde{\mathbf{X}}_j\|\,\|\widetilde{\mathbf{a}}\|} + \frac{\|\widetilde{\mathbf{a}}\|}{\|\widetilde{\mathbf{Y}}_j\|}.$$

We need to prove that the first term on the RHS has negative expected value. Let us decompose this term into the positive and negative parts,

$$\frac{\|\widetilde{\mathbf{X}}_j\|}{\|\widetilde{\mathbf{X}}_j + \widetilde{\mathbf{a}}\|}\,\frac{\widetilde{\mathbf{X}}_j'\,\widetilde{\mathbf{a}}}{\|\widetilde{\mathbf{X}}_j\|\,\|\widetilde{\mathbf{a}}\|} = \left(\frac{\|\widetilde{\mathbf{X}}_j\|}{\|\widetilde{\mathbf{X}}_j + \widetilde{\mathbf{a}}\|}\,\frac{\widetilde{\mathbf{X}}_j'\,\widetilde{\mathbf{a}}}{\|\widetilde{\mathbf{X}}_j\|\,\|\widetilde{\mathbf{a}}\|}\right)^+ - \left(\frac{\|\widetilde{\mathbf{X}}_j\|}{\|\widetilde{\mathbf{X}}_j + \widetilde{\mathbf{a}}\|}\,\frac{\widetilde{\mathbf{X}}_j'\,\widetilde{\mathbf{a}}}{\|\widetilde{\mathbf{X}}_j\|\,\|\widetilde{\mathbf{a}}\|}\right)^-,$$

where $X^+ = \max(X,0)$ and $X^- = -\min(X,0)$.

Because $\|\widetilde{\mathbf{X}}_j + \widetilde{\mathbf{a}}\|^2 = \|\widetilde{\mathbf{X}}_j\|^2 + \|\widetilde{\mathbf{a}}\|^2 + 2\widetilde{\mathbf{X}}_j'\widetilde{\mathbf{a}}$,

$$\left(\frac{\|\widetilde{\mathbf{X}}_j\|}{\|\widetilde{\mathbf{X}}_j + \widetilde{\mathbf{a}}\|}\,\frac{\widetilde{\mathbf{X}}_j'\,\widetilde{\mathbf{a}}}{\|\widetilde{\mathbf{X}}_j\|\,\|\widetilde{\mathbf{a}}\|}\right)^+ \leq \left(\frac{\|\widetilde{\mathbf{X}}_j\|}{\sqrt{\|\widetilde{\mathbf{X}}_j\|^2 + \|\widetilde{\mathbf{a}}\|^2}}\,\frac{\widetilde{\mathbf{X}}_j'\,\widetilde{\mathbf{a}}}{\|\widetilde{\mathbf{X}}_j\|\,\|\widetilde{\mathbf{a}}\|}\right)^+,$$

$$\left(\frac{\|\widetilde{\mathbf{X}}_j\|}{\|\widetilde{\mathbf{X}}_j + \widetilde{\mathbf{a}}\|}\,\frac{\widetilde{\mathbf{X}}_j'\,\widetilde{\mathbf{a}}}{\|\widetilde{\mathbf{X}}_j\|\,\|\widetilde{\mathbf{a}}\|}\right)^- \geq \left(\frac{\|\widetilde{\mathbf{X}}_j\|}{\sqrt{\|\widetilde{\mathbf{X}}_j\|^2 + \|\widetilde{\mathbf{a}}\|^2}}\,\frac{\widetilde{\mathbf{X}}_j'\,\widetilde{\mathbf{a}}}{\|\widetilde{\mathbf{X}}_j\|\,\|\widetilde{\mathbf{a}}\|}\right)^-.$$

These inequalities are identities when $\widetilde{\mathbf{X}}_j'\,\widetilde{\mathbf{a}}$ is of opposite sign to $(\,\cdot\,)^{\pm}$, or when $\widetilde{\mathbf{X}}_j'\,\widetilde{\mathbf{a}} = 0$. Because of the permutation symmetries of $\widetilde{\mathbf{X}}_j/\|\widetilde{\mathbf{X}}_j\|$, it follows that $\mathrm{P}(\,\widetilde{\mathbf{X}}_j'\,\widetilde{\mathbf{a}} \neq 0\,) > 0$,

which implies

$$\mathrm{P}\left(\left(\frac{\|\widetilde{\mathbf{X}}_j\|}{\|\widetilde{\mathbf{X}}_j+\widetilde{\mathbf{a}}\|}\,\frac{\widetilde{\mathbf{X}}_j'\,\widetilde{\mathbf{a}}}{\|\widetilde{\mathbf{X}}_j\|\,\|\widetilde{\mathbf{a}}\|}\right)^{+} \;<\; \left(\frac{\|\widetilde{\mathbf{X}}_j\|}{\sqrt{\|\widetilde{\mathbf{X}}_j\|^2+\|\widetilde{\mathbf{a}}\|^2}}\,\frac{\widetilde{\mathbf{X}}_j'\,\widetilde{\mathbf{a}}}{\|\widetilde{\mathbf{X}}_j\|\,\|\widetilde{\mathbf{a}}\|}\right)^{+}\right) \;>\; 0,$$

$$\mathrm{P}\left(\left(\frac{\|\widetilde{\mathbf{X}}_j\|}{\|\widetilde{\mathbf{X}}_j+\widetilde{\mathbf{a}}\|}\,\frac{\widetilde{\mathbf{X}}_j'\,\widetilde{\mathbf{a}}}{\|\widetilde{\mathbf{X}}_j\|\,\|\widetilde{\mathbf{a}}\|}\right)^{-} \;>\; \left(\frac{\|\widetilde{\mathbf{X}}_j\|}{\sqrt{\|\widetilde{\mathbf{X}}_j\|^2+\|\widetilde{\mathbf{a}}\|^2}}\,\frac{\widetilde{\mathbf{X}}_j'\,\widetilde{\mathbf{a}}}{\|\widetilde{\mathbf{X}}_j\|\,\|\widetilde{\mathbf{a}}\|}\right)^{-}\right) \;>\; 0,$$

and hence

$$\mathrm{E}\left(\left(\frac{\|\widetilde{\mathbf{X}}_j\|}{\|\widetilde{\mathbf{X}}_j+\widetilde{\mathbf{a}}\|}\,\frac{\widetilde{\mathbf{X}}_j'\,\widetilde{\mathbf{a}}}{\|\widetilde{\mathbf{X}}_j\|\,\|\widetilde{\mathbf{a}}\|}\right)^{+}\right) < \mathrm{E}\left(\left(\frac{\|\widetilde{\mathbf{X}}_j\|}{\sqrt{\|\widetilde{\mathbf{X}}_j\|^2+\|\widetilde{\mathbf{a}}\|^2}}\,\frac{\widetilde{\mathbf{X}}_j'\,\widetilde{\mathbf{a}}}{\|\widetilde{\mathbf{X}}_j\|\,\|\widetilde{\mathbf{a}}\|}\right)^{+}\right),$$

$$\mathrm{E}\left(\left(\frac{\|\widetilde{\mathbf{X}}_j\|}{\|\widetilde{\mathbf{X}}_j+\widetilde{\mathbf{a}}\|}\,\frac{\widetilde{\mathbf{X}}_j'\,\widetilde{\mathbf{a}}}{\|\widetilde{\mathbf{X}}_j\|\,\|\widetilde{\mathbf{a}}\|}\right)^{-}\right) > \mathrm{E}\left(\left(\frac{\|\widetilde{\mathbf{X}}_j\|}{\sqrt{\|\widetilde{\mathbf{X}}_j\|^2+\|\widetilde{\mathbf{a}}\|^2}}\,\frac{\widetilde{\mathbf{X}}_j'\,\widetilde{\mathbf{a}}}{\|\widetilde{\mathbf{X}}_j\|\,\|\widetilde{\mathbf{a}}\|}\right)^{-}\right).$$

For any permutation matrix $P \in \mathcal{P}_n$,

$$\frac{\|\widetilde{\mathbf{X}}_j\|}{\sqrt{\|\widetilde{\mathbf{X}}_j\|^2+\|\widetilde{\mathbf{a}}\|^2}} \;=\; \frac{\|P\,\widetilde{\mathbf{X}}_j\|}{\sqrt{\|P\,\widetilde{\mathbf{X}}_j\|^2+\|\widetilde{\mathbf{a}}\|^2}} \quad \text{surely,}$$

$$\frac{\widetilde{\mathbf{X}}_j}{\|\widetilde{\mathbf{X}}_j\|} \;=\; P\,\frac{\widetilde{\mathbf{X}}_j}{\|\widetilde{\mathbf{X}}_j\|} \quad \text{in distribution,}$$

so that

$$\frac{\|\widetilde{\mathbf{X}}_j\|}{\sqrt{\|\widetilde{\mathbf{X}}_j\|^2+\|\widetilde{\mathbf{a}}\|^2}}\,\frac{\widetilde{\mathbf{X}}_j}{\|\widetilde{\mathbf{X}}_j\|} \;=\; P\,\frac{\|\widetilde{\mathbf{X}}_j\|}{\sqrt{\|\widetilde{\mathbf{X}}_j\|^2+\|\widetilde{\mathbf{a}}\|^2}}\,\frac{\widetilde{\mathbf{X}}_j}{\|\widetilde{\mathbf{X}}_j\|} \quad \text{in distribution,}$$

which together with

$$\left(\frac{\|\widetilde{\mathbf{X}}_j\|}{\sqrt{\|\widetilde{\mathbf{X}}_j\|^2+\|\widetilde{\mathbf{a}}\|^2}}\,\frac{\widetilde{\mathbf{X}}_j}{\|\widetilde{\mathbf{X}}_j\|}\right)'\widehat{\mathbf{1}} \;=\; 0 \quad \text{surely,}$$

implies, as in (45), that

$$\mathrm{E}\left(\frac{\|\widetilde{\mathbf{X}}_j\|}{\sqrt{\|\widetilde{\mathbf{X}}_j\|^2+\|\widetilde{\mathbf{a}}\|^2}}\,\frac{\widetilde{\mathbf{X}}_j}{\|\widetilde{\mathbf{X}}_j\|}\right) \;=\; \mathbf{0}.$$

Therefore,

$$\mathrm{E}\left(\left(\frac{\|\widetilde{\mathbf{X}}_j\|}{\sqrt{\|\widetilde{\mathbf{X}}_j\|^2+\|\widetilde{\mathbf{a}}\|^2}}\frac{\widetilde{\mathbf{X}}_j'\,\widetilde{\mathbf{a}}}{\|\widetilde{\mathbf{X}}_j\|\,\|\widetilde{\mathbf{a}}\|}\right)^{+}\right) \;=\; \mathrm{E}\left(\left(\frac{\|\widetilde{\mathbf{X}}_j\|}{\sqrt{\|\widetilde{\mathbf{X}}_j\|^2+\|\widetilde{\mathbf{a}}\|^2}}\frac{\widetilde{\mathbf{X}}_j'\,\widetilde{\mathbf{a}}}{\|\widetilde{\mathbf{X}}_j\|\,\|\widetilde{\mathbf{a}}\|}\right)^{-}\right).$$

Back to the initial expected values, it follows that

$$\mathrm{E}\left(\left(\frac{\|\widetilde{\mathbf{X}}_j\|}{\|\widetilde{\mathbf{X}}_j+\widetilde{\mathbf{a}}\|}\frac{\widetilde{\mathbf{X}}_j'\,\widetilde{\mathbf{a}}}{\|\widetilde{\mathbf{X}}_j\|\,\|\widetilde{\mathbf{a}}\|}\right)^{+}\right) \;<\; \mathrm{E}\left(\left(\frac{\|\widetilde{\mathbf{X}}_j\|}{\|\widetilde{\mathbf{X}}_j+\widetilde{\mathbf{a}}\|}\frac{\widetilde{\mathbf{X}}_j'\,\widetilde{\mathbf{a}}}{\|\widetilde{\mathbf{X}}_j\|\,\|\widetilde{\mathbf{a}}\|}\right)^{-}\right),$$

which implies

$$\mathrm{E}\left(\frac{\|\widetilde{\mathbf{X}}_j\|}{\|\widetilde{\mathbf{X}}_j+\widetilde{\mathbf{a}}\|}\frac{\widetilde{\mathbf{X}}_j'\,\widetilde{\mathbf{a}}}{\|\widetilde{\mathbf{X}}_j\|\,\|\widetilde{\mathbf{a}}\|}\right) \;<\; 0. \quad \square$$

The Gaussian multivariate distribution, among others, has spherical symmetry besides permutation symmetry. For parametric families with spherical symmetry, the true standard vector $\sqrt{n-1}\,\widetilde{\mathbf{X}}_j/\|\widetilde{\mathbf{X}}_j\|$ has uniform distribution over the $(n-2)$-sphere. As a result, the components of $\widetilde{\mathbf{Y}}_j/\|\widetilde{\mathbf{Y}}_j\|$ perpendicular to $\widetilde{\mathbf{a}}$ are antisymmetric with respect to the direction of $\widetilde{\mathbf{a}}$, so that they cancel out in expectation. That is, for parametric families with spherical symmetry, and as long as $\widetilde{\mathbf{a}} \neq \mathbf{0}$,

$$\mathrm{E}\left(\frac{\widetilde{\mathbf{Y}}_j}{\|\widetilde{\mathbf{Y}}_j\|}\right) \;=\; \lambda\widetilde{\mathbf{a}}, \quad \text{with} \quad 0<\lambda<\mathrm{E}\left(\frac{1}{\|\widetilde{\mathbf{Y}}_j\|}\right). \tag{48}$$

4.4 Standard-vector normalization

The properties (47), (48) suggest the use of

$$\widehat{\mathbf{b}} \;=\; \frac{\displaystyle\sum_{j=1}^{m}\frac{\widetilde{\mathbf{y}}_j}{\|\widetilde{\mathbf{y}}_j\|}}{\displaystyle\sum_{j=1}^{m}\frac{1}{\|\widetilde{\mathbf{y}}_j\|}} \tag{49}$$

to approximate the unknown residual vector of normalization factors $\widetilde{\mathbf{a}}$. The following iterative method implements this approach to solve the *normalization of residuals problem.*

Let us define the following recursive sequence, where each step t comprises m vectors $\widehat{\mathbf{y}}_j^{(t)}$

($j \in \{1, \ldots, m\}$) and one vector $\widehat{\mathbf{b}}^{(t)}$,

$$\begin{aligned}
\widehat{\mathbf{y}}_j^{(0)} &= \widetilde{\mathbf{y}}_j, && (50)\\
\widehat{\mathbf{y}}_j^{(t)} &= \widehat{\mathbf{y}}_j^{(t-1)} - \widehat{\mathbf{b}}^{(t-1)}, \quad \text{for } t \geq 1, && (51)\\
\widehat{\mathbf{b}}^{(t)} &= \frac{\displaystyle\sum_{i=1}^{m} \frac{\widehat{\mathbf{y}}_j^{(t)}}{\|\widehat{\mathbf{y}}_j^{(t)}\|}}{\displaystyle\sum_{i=1}^{m} \frac{1}{\|\widehat{\mathbf{y}}_j^{(t)}\|}}, \quad \text{for } t \geq 0. && (52)
\end{aligned}$$

We assume that $\widehat{\mathbf{y}}_j^{(t)} \neq \mathbf{0}_n$, for all $j \in \{1, \ldots, m\}$ and all $t \geq 0$. Nonetheless, an implementation of this algorithm benefits from trimming out a small fraction (e.g. 1%) of the features with lesser $\|\widehat{\mathbf{y}}_j^{(t)}\|$ in (52), in order to avoid numerical singularities.

Let us write $\widehat{\mathbf{y}}_j^{(t)}$ as a function of the unknowns $\widetilde{\mathbf{x}}_j$ and $\widetilde{\mathbf{a}}$. For any $t \geq 1$,

$$\begin{aligned}
\widehat{\mathbf{y}}_j^{(t)} &= \widehat{\mathbf{y}}_j^{(t-1)} - \widehat{\mathbf{b}}^{(t-1)}, && (53)\\
&= \widehat{\mathbf{y}}_j^{(t-2)} - \widehat{\mathbf{b}}^{(t-2)} - \widehat{\mathbf{b}}^{(t-1)}, && (54)\\
&\vdots && (55)\\
&= \widehat{\mathbf{y}}_j^{(0)} - \sum_{r=0}^{t-1} \widehat{\mathbf{b}}^{(r)}, && (56)\\
&= \widetilde{\mathbf{y}}_j - \sum_{r=0}^{t-1} \widehat{\mathbf{b}}^{(r)}, && (57)\\
&= \widetilde{\mathbf{x}}_j + \widetilde{\mathbf{a}} - \sum_{r=0}^{t-1} \widehat{\mathbf{b}}^{(r)}. && (58)
\end{aligned}$$

Note that (58) is also valid for $t = 0$.

Let us also define the vectors $\widehat{\mathbf{a}}^{(t)}$, for $t \geq 0$, which describe the vector of normalization factors still to be removed at step t,

$$\widehat{\mathbf{a}}^{(t)} = \widetilde{\mathbf{a}} - \sum_{r=0}^{t-1} \widehat{\mathbf{b}}^{(r)}, \tag{59}$$

so that, by (58), for $t \geq 0$,

$$\widehat{\mathbf{y}}_j^{(t)} = \widetilde{\mathbf{x}}_j + \widehat{\mathbf{a}}^{(t)}. \tag{60}$$

Therefore, the recursive sequence (50)–(52) faces a new, weaker *normalization of residuals problem* at each step t, with true residual vectors $\widetilde{\mathbf{x}}_j$, observed residual vectors $\widehat{\mathbf{y}}_j^{(t)}$ and unknown normalization factors $\widehat{\mathbf{a}}^{(t)}$. The step t results in the estimation of normalization factors $\widehat{\mathbf{b}}^{(t)}$, which are removed from $\widehat{\mathbf{y}}_j^{(t)}$, generating the next step. At the beginning, $\widehat{\mathbf{y}}_j^{(0)} = \widetilde{\mathbf{y}}_j$ and $\widehat{\mathbf{a}}^{(0)} = \widetilde{\mathbf{a}}$.

At convergence, $\lim_{t\to\infty} \widehat{\mathbf{b}}^{(t)} = \mathbf{0}$. Equations (45), (46), (52) imply that, in such a case, $\lim_{t\to\infty} \widehat{\mathbf{y}}_j^{(t)} = \widetilde{\mathbf{x}}_j$ and $\sum_{t=0}^{\infty} \widehat{\mathbf{b}}^{(t)} = \widetilde{\mathbf{a}}$. Convergence is optimal when the parametric family of the m features has spherical symmetry, Gaussian being the most prominent case. Otherwise, the more uniform the distribution of standard vectors $\sqrt{n-1}\,\widetilde{\mathbf{x}}_j/\|\widetilde{\mathbf{x}}_j\|$ is on the $(n-2)$-sphere, the faster the sequence (50)–(52) converges. See examples of convergence in Supplementary Movies S1–S3.

4.5 Identification of no-variation genes

Let us consider a gene expression dataset, with g genes and c experimental conditions. Each condition k has s_k samples. The total number of samples is $s = \sum_{k=1}^{c} s_k$. Let us assume that $c \geq 2$ and that $s_k \geq 2$, for all conditions $k \in \{1, \ldots, c\}$. Let us also assume that, among the g genes, there is a fraction π_0 of non-differentially expressed genes (non-DEGs), with $0 \leq \pi_0 \leq 1$, while the remaining fraction $1 - \pi_0$ comprises the differentially expressed genes (DEGs)[5].

Let us consider the usual ANOVA test comparing average expression levels across conditions, gene-by-gene. Under the null hypothesis of a non-DEG, the corresponding F-statistic follows the F-distribution with $c-1$ and $s-c$ degrees of freedom. The test of this hypothesis yields a p-value p_j for each gene $j \in \{1, \ldots, g\}$. The obtained p-values p_j follow a probability distribution that can be considered as the mixture of two probability distributions, F_0 and F_1, for the non-DEGs and the DEGs, respectively[6]. The fraction π_0 of non-DEGs follows the uniform distribution on the interval $[0, 1]$,

$$F_0(p) \;=\; p, \tag{61}$$

while the fraction $1-\pi_0$ of DEGs follows a distribution that verifies, for any $p \in (0,1)$,

$$F_1(p) \; > \; p, \tag{62}$$

and the mixture distribution is

$$F(p) \; = \; \pi_0 F_0(p) + (1-\pi_0) F_1(p). \tag{63}$$

Let us further assume that there exists a p^*, with $0 < p^* < 1$, such that $F_1(p) = 1$ for every $p \geq p^*$. In other words, all DEGs have p-value p_j from the ANOVA test such that $p_j \leq p^*$, while only some genes among the non-DEGs have p-value with $p_j > p^*$. This implies that the mixture distribution of p-values is uniform on the interval $[p^*, 1]$,

$$F(p) \; = \; \pi_0 p + 1 - \pi_0, \quad \text{for } p^* \leq p \leq 1, \tag{64}$$

$$f(p) \; = \; \pi_0, \quad \text{for } p^* < p < 1. \tag{65}$$

On the other hand, for any set of n samples $x_{(1)} \leq x_{(2)} \leq \cdots \leq x_{(n)}$ obtained from n independent and identically distributed uniform random variables on the interval $[a,b]$, all the distances between consecutive ordered samples (including boundaries), $x_{(1)} - a, x_{(2)} - x_{(1)}, \ldots, x_{(n)} - x_{(n-1)}, b - x_{(n)}$, obey the same distribution[7]. Then, it can be realized that, for any j such that $2 \leq j \leq n-1$, the two subsets of samples $x_{(1)}, \ldots, x_{(j-1)}$ and $x_{(j+1)}, \ldots, x_{(n)}$ follow uniform distributions on the intervals $[a, x_{(j)}]$ and $[x_{(j)}, b]$, respectively.

Based on these facts, to identify no-variation genes we propose finding the minimum $p_{(j)}$, from the ordered sequence of p-values $p_{(1)} \leq p_{(2)} \leq \cdots \leq p_{(g)}$, such that a goodness-of-fit test for the uniform distribution on the interval $[p_{(j)}, 1]$, performed on $p_{(j+1)}, \ldots, p_{(g)}$, is not rejected. As a result, the genes corresponding to the p-values $p_{(j)}, p_{(j+1)}, \ldots, p_{(g)}$ are considered as no-variation genes.

Given the concavity of $F(p)$, the goodness-of-fit test used is the one-sided Kolmogorov-Smirnov test on positive deviations of the empirical distribution function.

See Supplementary Movies S4–S6 for examples of this approach to identifying no-variation genes.

References

[1] Eaton, M. L. *Multivariate Statistics: A Vector Space Approach* (Institute of Mathematical Statistics, Beachwood, Ohio, 2007).

[2] Fang, K., Kotz, S. & Ng, K. W. *Symmetric Multivariate and Related Distributions* (Chapman and Hall, New York, 1990).

[3] Gupta, A. K., Varga, T. & Bodnar, T. *Elliptically Contoured Models in Statistics and Portfolio Theory* (Springer, New York, 2013).

[4] Kallenberg, O. *Probabilistic Symmetries and Invariance Principles* (Springer, New York, 2005).

[5] Storey, J. D. & Tibshirani, R. Statistical significance for genomewide studies. *Proc Natl Acad Sci USA* **100**, 9440–9445 (2003).

[6] Storey, J. D. The positive false discovery rate: a bayesian interpretation and the q-value. *Ann Stat* **31**, 2013–2035 (2003).

[7] Feller, W. *An Introduction to Probability Theory and Its Applications*, vol. 2 (Wiley, New York, 1971), 2 edn.
